# Supplementary material for: Positron emission tomography and magnetic resonance imaging in experimental human malaria to identify organ-specific changes in morphology and glucose metabolism: A prospective cohort study
Source: PLoS Med. 2021 May 26;18(5):e1003567. doi: 10.1371/journal.pmed.1003567 (PMC8154100; doi:10.1371/journal.pmed.1003567)
Supplement: S1 Text — (PDF) [file pmed.1003567.s002.pdf]

S1 Text: Methods

Magnetic Resonance Imaging

Splenic sequences included inspiratory and expiratory T1 weighted acquisitions and Dixon sequences for PET attenuation correction. The remainder of the body underwent T1 weighted and T2 turbo spin echo (TSE) sequences. No contrast medium was administered.

Spleen	Whole body
• Dixon PET attenuation scan<br>3x Dixon images at expiration, inspiration, and mid-exhale (unused in presented analysis)<br>• PET Acquisition<br>• 2D Half-Fourier-Acquired Single-shot Turbo spin Echo (HASTE) images (unused in presented analysis)	• PET Acquisition<br>• Dixon PET attenuation scan<br>• T2 TSE<br>• T1 Volumetric interpolated breath-hold examination (VIBE)

Positron Emission Tomography

Participants were administered a standard dosage of approximately 4.0MBq/kg of the radiotracer 18-F fluorodeoxyglucose (FDG) prior to PET imaging. Radiotracer was manufactured on site at the Royal Brisbane and Women's Hospital Department of Nuclear Medicine as per Good Manufacturing Practice. Dynamic, quantitative radiotracer uptake was recorded over a 45-minute period across an abdominal field of view encompassing the spleen, liver, and vertebral bone marrow. Images were reconstructed to 4.2x4.2x2.0 mm^3 (LRxAPxSI) resolution in 20 dynamic time frames (8x 15s, 2x 30s, 2x 60s, 8x 300s) using ordered-subsets expectation maximization with corrections for random events, scatter, normalization, attenuation, decay, and partial volume (HD-PET, Siemens, USA). Static, semi-quantitative radiotracer uptake

using standardized uptake values (SUVs) was recorded for the whole body. Gamma-photon PET attenuation correction was performed using 4-class segmentation of a Dixon MR sequence [1].

Imaging Analysis

Pre-specified abdominal regions of interest (ROIs) included the spleen, vertebral bone marrow and liver. Reporting clinicians were blinded to the challenge agent species. Due to electronic date stamping of all imaging data, baseline and post-inoculation episodes were not blinded. Post-inoculation imaging was reviewed with respect to baseline imaging. An external radiologist and a nuclear medicine physician reviewed all MRI and PET imaging for incidental findings and safety assessment as per local guidelines.

Abdominal ROIs were contoured manually using ITK-SNAP 3.2, Pennsylvania, USA (PJ). Quantitative splenic and liver volume data were calculated from these contours. The body of the third lumbar vertebra was chosen as representative of the vertebral bone marrow as it was readily examined in the same imaging field as the abdominal organs. Semi-quantitative SUVs were estimated for the pre-specified abdominal ROIs. A further exploratory quantitative analysis was conducted on the dynamic acquisition, using the Gjedde-Patlak graphical technique [2], where steady-state was assumed to have been met after 5 minutes [3]. The output of this analysis was the uptake rate of radiotracer into tissue from the plasma (Ki) in each of the abdominal ROIs except the liver, where the irreversible uptake assumption of the model was not satisfied (AG).

Study Safety

All participants were assessed for standard induced blood stage malaria (IBSM) study inclusion criteria. Participants were administered curative antimalarial treatment following the development of clinical signs of malaria, or after reaching a pre-specified threshold parasitemia. Standard safety assessments including clinical review, hematology and biochemistry parameters were performed at specified times during the IBSM studies including baseline, one day following post-inoculation imaging (at time of treatment), and in convalescence 1-2 weeks after post-inoculation imaging. Due to differences in the contributing IBSM studies, not all participants had safety assessments on the same days relative to imaging.

S1 Text: References

1. Martinez-Moller A, Souvatzoglou M, Delso G, Bundschuh RA, Chefd'hotel C, Ziegler SI, et al. Tissue classification as a potential approach for attenuation correction in whole-body PET/MRI: evaluation with PET/CT data. Journal of nuclear medicine : official publication, Society of Nuclear Medicine. 2009;50(4):520-6. doi: 10.2967/jnumed.108.054726. PubMed PMID: 19289430.
2. Gjedde A. Calculation of cerebral glucose phosphorylation from brain uptake of glucose analogs in vivo: a re-examination. Brain Res. 1982;257(2):237-74. doi: 10.1016/0165-0173(82)90018-2. PubMed PMID: 7104768.
3. Patlak CS, Blasberg RG, Fenstermacher JD. Graphical evaluation of blood-to-brain transfer constants from multiple-time uptake data. Journal of cerebral blood flow and metabolism : official journal of the International Society of Cerebral Blood Flow and Metabolism. 1983;3(1):1-7. doi: 10.1038/jcbfm.1983.1. PubMed PMID: 6822610.
