# Supplementary material for: Positron emission tomography and magnetic resonance imaging in experimental human malaria to identify organ-specific changes in morphology and glucose metabolism: A prospective cohort study
Source: PLoS Med. 2021 May 26;18(5):e1003567. doi: 10.1371/journal.pmed.1003567 (PMC8154100; doi:10.1371/journal.pmed.1003567)
Supplement: S2 Text — (PDF) [file pmed.1003567.s003.pdf]

S2 Text: Results

Quantitative radiotracer uptake in regions of interest

As an exploratory further analysis, the rate of irreversible radiotracer uptake was modelled using a Patlak analysis for the ROIs. As some of these results approach zero, percentage change from baseline has not been presented (S1 Table).

There was no significant change in splenic Ki uptake rate from baseline for *P. vivax* (mean 0.002min^{-1} [95% CI -0.001 to 0.004] vs 0.003min^{-1} [CI -0.001 to 0.007], paired t-test p=0.28) or for *P. falciparum* (0.001min^{-1} [range -0.001 to 0.002] vs. 0.003min^{-1} [CI -0.002 to 0.009], paired t-test p=0.22). There was no difference between groups (unpaired t-test, p=0.72). Similarly, there was no significant change in vertebral bone marrow Ki uptake rates from baseline for *P. vivax* (median 0.006min^{-1} [CI 0.004 to 0.008] vs. 0.005min^{-1} [CI 0.001 to 0.009], paired t-test p=0.71) or for *P. falciparum* (0.004min^{-1} [CI 0.003 to 0.006) vs. 0.006min^{-1} (range 0.003 to 0.008), paired t-test p=0.07). There was no difference between groups (unpaired t-test p=0.23). Both spleen and vertebral bone marrow Ki uptake rate and change in SUV from baseline to post-inoculation imaging were not correlated for either group (*P. viva*x: spleen Spearman r=0.50, p=1.00 and vertebral bone marrow Spearman r=0.50, p=1.00; *P. falciparum:* spleen Spearman r=0.40, p=0.75, vertebral bone marrow Spearman r=0.60, p=0.42). This is consistent with previous studies, which have found a poor correlation between SUV and Patlak modelled radiotracer functions [1]. This may be related to SUV primarily reflecting blood pool activity, whereas uptake modelling reflects tissue-based phosphorylation.

Quantitative uptake could not be reliably calculated for the liver using our modelling. This may be related to de-phosphorylation of radiotracer by hepatic glucose-6-phosphatase causing reversibility of uptake and violating the assumptions of the Patlak model [2]. Furthermore, the dual blood supply to the liver (systemic and portal circulation) may also influence the interpretation of results, where dual input modelling and invasive blood sampling beyond the scope of this investigation may improve accuracy [3].

Radiotracer uptake variability

Intrinsic factors such as age, gender and body mass index, and extrinsic factors such as blood glucose and imaging uptake time may affect tissue radiotracer uptake [4]. In this study, variability between baseline and post-inoculation imaging not related to malaria inoculation has been controlled as much as reasonably possible. The use of paired baseline and post-inoculation imaging eliminated variability in intrinsic factors. Furthermore, by screening for impaired fasting glucose at enrolment and keeping similar fasted conditions between scans with matched baseline and post-inoculation radiotracer uptake times, extrinsic factors were also controlled. At an individual level, all participants were of a similar age, and gender and body mass index did not appear to be associated with extremes in either absolute SUVs or change in SUVs from baseline.

S2 Text: References

1. Keramida G, Anagnostopoulos CD, Peters AM. The extent to which standardized uptake values reflect FDG phosphorylation in the liver and spleen as functions of time after injection of (18)F-fluorodeoxyglucose. EJNMMI Res.

2017;7(1):13. doi: 10.1186/s13550-017-0254-7. PubMed PMID: 28176243; PubMed Central PMCID: PMCPMC5296268.
2. Caraco C, Aloj L, Chen LY, Chou JY, Eckelman WC. Cellular release of [18F]2-fluoro-2-deoxyglucose as a function of the glucose-6-phosphatase enzyme system. J Biol Chem. 2000;275(24):18489-94. doi: 10.1074/jbc.M908096199. PubMed PMID: 10764804.
3. Munk OL, Bass L, Roelsgaard K, Bender D, Hansen SB, Keiding S. Liver kinetics of glucose analogs measured in pigs by PET: importance of dual-input blood sampling. Journal of nuclear medicine : official publication, Society of Nuclear Medicine. 2001;42(5):795-801. PubMed PMID: 11337579.
4. Wang R, Chen H, Fan C. Impacts of time interval on 18F-FDG uptake for PET/CT in normal organs: A systematic review. Medicine (Baltimore). 2018;97(45):e13122. doi: 10.1097/MD.0000000000013122. PubMed PMID: 30407330; PubMed Central PMCID: PMCPMC6250532.
