# Supplementary material for: Positron emission tomography and magnetic resonance imaging in experimental human malaria to identify organ-specific changes in morphology and glucose metabolism: A prospective cohort study
Source: PLoS Med. 2021 May 26;18(5):e1003567. doi: 10.1371/journal.pmed.1003567 (PMC8154100; doi:10.1371/journal.pmed.1003567)
Supplement: S1 Table — (PDF) [file pmed.1003567.s004.pdf]

S1 Table: Abdominal quantitative imaging metrics. SUV: standardized uptake value (no units), Ki: quantitative rate of radiotracer uptake (min^{-1}), BL: baseline, PI: post-inoculation, %: percentage change from baseline

Challenge agent	Participant	Splenic Volume (mL)			Splenic SUV			Splenic Ki (min^{-1})		Liver Volume (mL)			Liver SUV			Vertebral Bone Marrow SUV			Vertebral Bone Marrow Ki (min^{-1})	
		BL	PI	%	BL	PI	%	BL	PI	BL	PI	%	BL	PI	%	BL	PI	%	BL	PI
P.vivax	1	261	308	18.0	2.2	2.6	15.9	0.001	0.005	1532	1537	0.3	3.3	3.4	1.9	1.4	1.4	1.0	0.007	0.003
	2	341	435	27.6	1.8	2.2	21.8	0.001	0.002	1805	1981	9.8	2.7	3.1	13.1	1.2	1.3	14.0	0.005	0.006
	3	287	404	40.8	2.0	2.2	8.8	0.003	0.003	1303	1181	-9.4	3.2	3.4	3.7	1.5	1.5	-1.2	0.005	0.006
P.falciparum	4	318	301	-5.4	2.2	2.4	7.7	0.001	0.002	1563	1514	-3.1	3.4	3.3	-4.2	1.1	1.1	-2.0	0.004	0.005
	5	206	242	17.6	2.5	2.6	1.9	0.001	0.000	1237	1243	0.5	3.9	3.8	-1.3	1.5	1.5	2.5	0.003	0.004
	6	181	277	52.7	1.9	2.0	6.7	0.000	0.006	1388	1536	10.7	2.7	2.7	0.0	1.0	1.1	7.6	0.005	0.006
	7	130	165	26.7	2.2	2.4	5.7	0.002	0.006	1450	1561	7.7	3.3	3.3	0.0	1.4	1.4	4.7	0.005	0.007
