# Supplementary material for: Positron emission tomography and magnetic resonance imaging in experimental human malaria to identify organ-specific changes in morphology and glucose metabolism: A prospective cohort study
Source: PLoS Med. 2021 May 26;18(5):e1003567. doi: 10.1371/journal.pmed.1003567 (PMC8154100; doi:10.1371/journal.pmed.1003567)
Supplement: S2 Table — (PDF) [file pmed.1003567.s005.pdf]

S2 Table: Hematology and biochemistry parameters. BL: baseline, PI: post-inoculation, C: convalescence

Challenge agent	Participant	Hemoglobin (g/L)			Hematocrit (L/L)			Platelets (10^9/L)			Total bilirubin (mmol/L)			Alanine Transaminase (IU/L)		
		BL	PI	C	BL	PI	C	BL	PI	C	BL	PI	C	BL	PI	C
P.vivax	1	127	117	111	0.38	0.34	0.32	222	123	218	8	9	6	13	16	86
	2	161	146	147	0.45	0.41	0.41	201	135	221	9	14	7	14	11	31
	3	155	148	142	0.45	0.42	0.41	196	143	215	13	22	17	18	22	28
P.falciparum	4	135	139	135	0.42	0.42	0.41	261	281	199	7	12	13	26	26	21
	5	149	152	135	0.45	0.43	0.39	194	162	180	7	6	7	31	32	85
	6	155	156	144	0.45	0.45	0.41	356	219	147	8	15	7	14	19	73
	7	145	144	144	0.43	0.41	0.43	317	265	177	5	7	7	21	19	128

Summary of statistical comparisons between laboratory parameters at different time points

**Parameter**	**Comparison (p value)**			
	***P. vivax* group**		***P. falciparum* group**	
	**BL vs. PI**	**PI vs. C**	**BL vs. PI**	**PI vs. C**
**Hemoglobin (g/L)**	0.045*	0.004*	0.213	0.162
**Hematocrit (L/L)**	0.008*	0.020*	0.182	0.140
**Platelets (10^9/L)**	0.034*	0.280	0.222	0.090
**Total bilirubin (mmol/L)**	0.163	0.999	0.160	0.340
**Alanine Transaminase (IU/L)**	0.604	0.237	0.546	0.101

Asterisk represents $p<0.05$ in paired two-tailed T-test comparison
