# Supplementary material for: Positron emission tomography and magnetic resonance imaging in experimental human malaria to identify organ-specific changes in morphology and glucose metabolism: A prospective cohort study
Source: PLoS Med. 2021 May 26;18(5):e1003567. doi: 10.1371/journal.pmed.1003567 (PMC8154100; doi:10.1371/journal.pmed.1003567)
Supplement: S1 Fig — (A) Hemoglobin (g/L), (B) Hematocrit (L/L), (C) Platelets (10^9/L), (D) Total Bilirubin (mmol/L), and (E) ALT (IU/L). (PDF) [file pmed.1003567.s006.pdf]

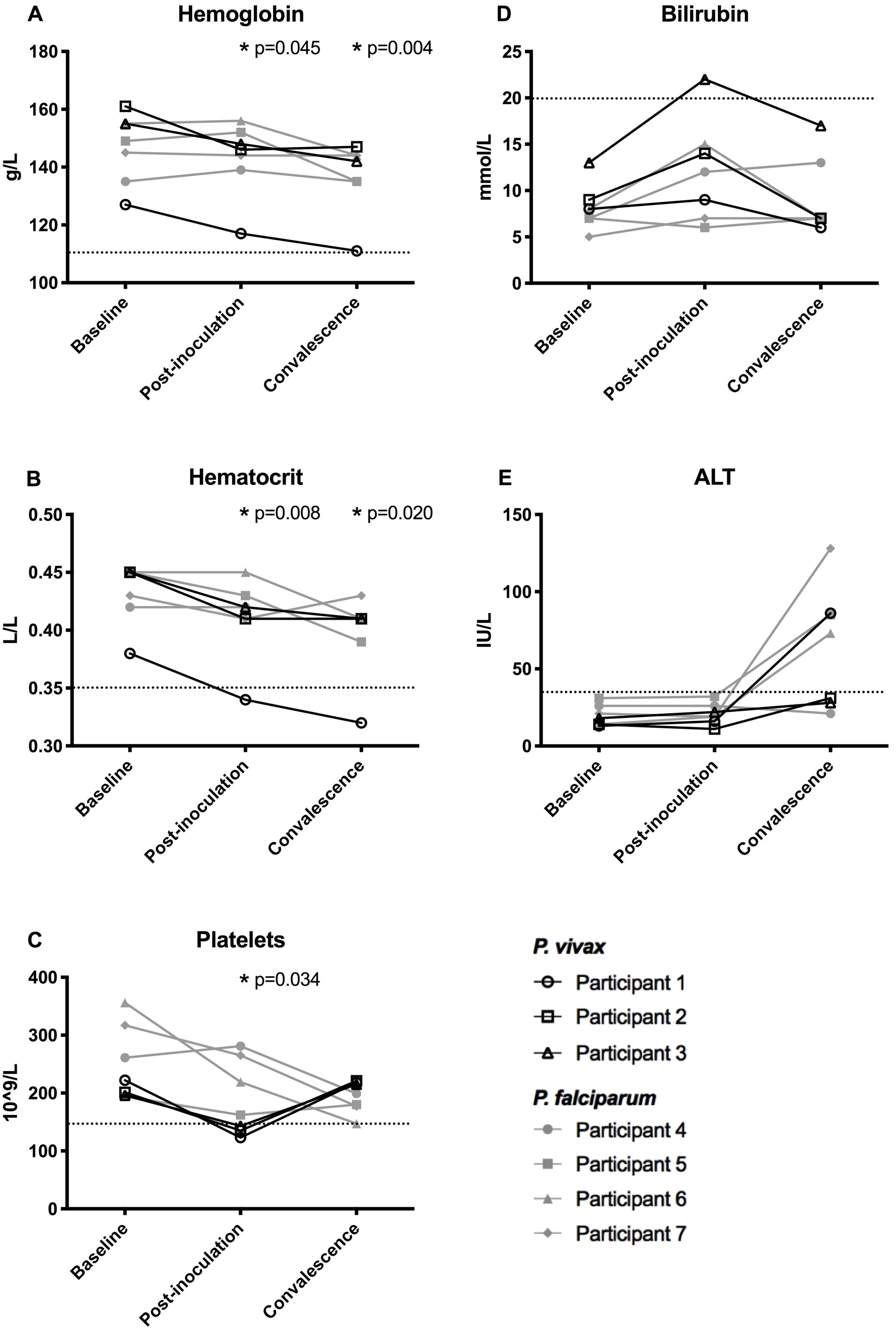

S1 Fig: Hematology and biochemistry parameter sat baseline, post-inoculation, and convalescence. A) Hemoglobin (g/L), B) Hematocrit (L/L), C) Platelets (10^9/L), D) Total Bilirubin (mmol/L), E) ALT (IU/L)

ALT: alanine transaminase

P. vivax group represented by black lines with unfilled markers

P. falciparum group represented by grey lines with filled markers

Asterisk represents p<0.05 in paired t-test comparison with baseline measurements (black for *P. vivax*, grey for *P. falciparum*)

Dotted line represents upper or lower limit of laboratory reference range
