# Supplementary material for: Positron emission tomography and magnetic resonance imaging in experimental human malaria to identify organ-specific changes in morphology and glucose metabolism: A prospective cohort study
Source: PLoS Med. 2021 May 26;18(5):e1003567. doi: 10.1371/journal.pmed.1003567 (PMC8154100; doi:10.1371/journal.pmed.1003567)
Supplement: S1 Protocol — (PDF) [file pmed.1003567.s007.pdf]

Exploratory Study Protocol

Functional nuclear medicine imaging in subpatent malaria: a pilot study

Protocol No: P2196
Version: 2.2
Date: 29September2016

For addition to the parallel IBSM MAIN study QP15C20 SJ733

ACTRN12616001458426 linked to IBSM study NCT02867059

PROTOCOL SYNOPSIS

Full Title: Functional nuclear medicine imaging in subpatent malaria: a pilot study

Short Title: NA

Project Number: P2196

Objectives:

Primary:

- To investigate the use of ^{18}F FDG-PET/MRI to estimate the biodistribution of *P. falciparum* in human volunteer induced blood stage malaria studies.
- To investigate the use of ^{18}F FDG-PET/MRI to estimate the biomass of *P. falciparum.*

Secondary:

- To describe the relative burden of organ-specific tissue sequestration in *P. falciparum.*
- To describe the impact of early malaria infection on glucose metabolism.
- To describe the MRI brain findings of early malaria infection in a prospective participant cohort.

Population: Two adult (male and female of non-childbearing potential) participants between 18 and 55 years of age, recruited from induced blood stage malaria (IBSM) model MAIN study populations (MAIN study refers to clinical trial QP15C20).

Phase: exploratory

Description of Investigation:

Participants will receive a whole body PET/MRI scan and dedicated brain MRI one to three days prior to inoculation (within roughly one week prior) and on one to two days prior to confinement with peak parasitaemia post BSP inoculation (estimated InD 6 to 7). Preparation involves fasting for 6 hours prior, abstaining from excessive physical exertion and following a low carbohydrate diet 24 hours prior (recorded on a diet and activity sheet). Scans will be performed on the Biograph mMR PET/MRI system after the intravenous infusion of the standard radiotracer 2-(^{18}F) fluor-deoxy-D-glucose(FDG) (^{18}F FDG) and MAGNETOM Prisma 3T MRI system. Collected images will be reviewed and reported by experienced radiologists specialising in MRI and nuclear medicine reporting. Quantification of ^{18}F FDG uptake measurements will be established for intra-individual scans using Patlak model analysis and semi quantitative SUV measurement with reference to an ^{18}F FDG external control.

Study Duration: October 2016 – June 2017

Participant Participation Duration: Estimated 8-14 days

Estimated Time to Complete Enrollment: August 2016 – March 2017

1. KEY ROLES AND CONTACT INFORMATION 9
  1.1 Study Location(s) 10
  1.2 Study Management 10
2. INTRODUCTION: BACKGROUND AND SCIENTIFIC RATIONALE 11
  2.1 Background Information 11
  2.2 Study Objectives 12
3. STUDY DESIGN 14
  3.1 Study Flow Chart 15
4. PARTICIPANT ENROLLMENT AND WITHDRAWAL 16
  4.1 Recruitment 16
  4.2 Eligibility Criteria 16
  4.3 Participant Withdrawal 17
  4.4 Permanent Termination or Suspension of Exploratory Study 17
5. STUDY INVESTIGATIONS 17
  5.1 Radiotracer Dosing Regimen 18
  5.2 Modification of Radiotracer Administration for a Participant 18
  5.3 Participant Compliance 18
  5.4 Radiotracer Manufacture, Handling and Accountability 18
6. STUDY SCHEDULE 19
  6.1 Screening 19
  6.2 Enrollment/Baseline 19
  6.3 Intermediate Visits 19
  6.4 Final Exploratory Study Visit 19
  6.5 Withdrawal Visit 19
  6.6 Unscheduled Visit 19
7. STUDY PROCEDURES AND EVALUATIONS 19
  7.1 Study Procedures 19
  7.2 Laboratory Procedures/Evaluations 20
8. ASSESSMENT OF SAFETY 21
  8.1 Specification of Safety Parameters 21
  8.2 Definition of an Adverse Event and of a Serious Adverse Event 21
  8.3 Documentation and classification of study specific adverse events 21
  8.4 Relationship to Study Investigation 22
  8.5 Recording and Prompt Reporting of Events 22
  8.6 Halting Rules 23
9. QUALITY ASSURANCE 23
10. STATISTICAL CONSIDERATIONS 23
  10.1 Sample Size 23

10.2 Statistical Analysis Plan 23

11. SOURCE DOCUMENTS AND ACCESS 24

12. ADMINISTRATIVE PROCEDURES 24

13. DATA HANDLING, RECORD KEEPING AND PUBLICATION POLICY 26

14. LITERATURE REFERENCES 27

15. APPENDICES 28

15.1 Appendix A: Radiation assessment Report 28

15.2 Appendix B: MRI checklist 29

15.3 Appendix C: Diet and Activity Sheet 30

15.4 Appendix D: Participant Information Sheet/Consent Form 31

15.5 Appendix E: HIRF site map 32

15.6 Appendix F: Licence to manufacture therapeutic goods 32

15.7 Appendix G: Product information 32

2. INTRODUCTION: BACKGROUND AND SCIENTIFIC RATIONALE

2.1 Background Information

Morbidity and mortality from malaria infection remains significant despite anti-parasitic treatments. Understanding the pathophysiology of disease may aid in the development of adjunctive therapies to improve survival in severe cases. Parasite sequestration in tissue microvasculature permits evasion of the reticulo-endothelial system and increased biomass [1]. Organ specific sequestration contributes to end organ dysfunction in severe malaria syndromes. Owing to the inaccessibility of sequestered parasites, estimations of total biomass and sites of sequestration have historically relied on biochemical markers [2-5], animal models [1, 6, 7] and post mortem studies [8]. Determination of the biodistribution of malaria with functional nuclear medicine imaging may allow for more direct study of sequestration in human models. This may aid developing a better understanding of the organ dysfunction experienced in severe cases.

Nuclear medicine imaging techniques, particular hybrid PET/CT and PET/MRI have a central role primarily in oncology for assessing the biodistribution and activity of malignancy. The ability of these techniques to detect and locate biological and biochemical changes have more recently been applied to other medical fields including Infectious Diseases, though there are no studies in malaria.

This EXPLORATORY STUDY has been designed as a prospective pilot investigation to establish the role of ^{18}F FDG-PET/MRI in studying the pathophysiology of subpatent malaria following low dose *Plasmodium falciparum* exposure in healthy adults. Collection of baseline and post inoculation imaging will provide information about changes in host/parasite glucose metabolism that may be used to estimate parasite biomass and biodistribution. Glucose uptake is increased up to 100-fold in parasitised erythrocytes [9], suggesting that ^{18}F FDG may be a viable radiotracer to help evaluate the disease.

Whole body PET/MRI is a functional imaging modality that provides detailed soft tissue anatomical information with lower ionizing radiation exposure compared to equivalent PET/CT. The radiotracer ^{18}F FDG is a safe and well-validated biomimetic for demonstrating glucose uptake. Imaging with ^{18}F FDG-PET/MRI is an ideal model for exploring nuclear medicine functional imaging in a healthy human volunteer population.

Biochemical estimates of parasite biomass and parasitaemia will be compared to quantified radiotracer uptake measurements. Comparison of interval changes in host/parasite glucose metabolism will add to our understanding of the metabolic changes associated with disease [10]. Although there have been several trials using MRI imaging to study malaria, none to date have been prospective. Comparison of MRI brain data with existing literature in uncomplicated malaria may help further describe the changes seen in early disease [11]. The feasibility of ^{18}F FDG-PET/MRI may contribute to developing a further role for nuclear medicine imaging in malaria.

Successful application of nuclear medicine imaging would provide a greater understanding of parasite sequestration dynamics, which may aid in disease modeling and development of treatments to prevent end organ damage in severe disease.

2.2 Study Objectives

Primary:

- To investigate the use of ^{18}F FDG-PET/MRI to estimate the biodistribution of *P. falciparum* in human volunteer induced blood stage malaria studies.
- To investigate the use of ^{18}F FDG-PET/MRI to estimate the biomass of *P. falciparum*.

Secondary:

- To describe the relative burden of organ-specific tissue sequestration in *P. falciparum*.
- To describe the impact of early malaria infection on glucose metabolism.
- To describe the MRI brain findings of early malaria infection in a prospective participant cohort.

i. Research Question

The present EXPLORATORY STUDY has been designed to establish the role of ^{18}F FDG-PET/MRI in studying the pathophysiology of subpatent malaria following low dose *Plasmodium falciparum* exposure in healthy adults.

ii. Study Outcome Measures

<u>Primary</u>

The primary objective of the study is to assess the application of whole body ^{18}F FDG-PET/MRI in describing the parasite biodistribution and biomass in subpatent malaria. This is a hypothesis generating pilot investigation expected to have predominately descriptive outcomes.

The primary outcome measures will be the quantified and semi-quantified ^{18}F FDG uptake values from specific regions of interest. Regions of interest for quantification of uptake will be spleen, bone marrow (lumbar spine and/or pelvis), muscle bulk (quadriceps) and brain. Post inoculation measurements will be compared to baseline pre inoculation uptake values. Biochemical markers of parasitaemia and total biomass from MAIN study data will be compared to ^{8}F FDG uptake values.

Any other regions of interested (as deemed by the investigators) identified after imaging will have semi-quantified ^{18}F FDG uptake values calculated for further comparison.

<u>Secondary</u>

The relative burden of organ specific sequestration will be determined by comparing ^{18}F FDG uptake values for each region of interest.

The impact of early malaria infection on glucose metabolism will be evaluated in assessing each of the above outcomes.

The MRI findings of early malaria will be presented in a descriptive manner, outlining any interval changes in high-resolution brain MRI for this prospective cohort.

iii. Study Rationale

This EXPLORATORY STUDY has been designed to establish the role of ^{18}F FDG-PET/MRI in studying the pathophysiology of subpatent malaria. The selection of this radiotracer and imaging modality offers a well-validated model for exploring nuclear medicine functional imaging of malaria infection in a healthy human volunteer population.

Hypotheses

- Whole body ^{18}F FDG-PET/MRI is a technically feasible imaging modality for the study of subpatent malaria infection.
- Quantitative and semi-quantitative uptake of ^{18}F FDG is proportional to estimated parasite biomass and parasitaemia.
- Subtle changes in the vasculature of the brain are present and identifiable on MRI imaging in subpatent malaria infection.

iv. Potential Risks and Benefits

<u>Potential Risks</u>

There is a small risk associated with radiation exposure from the radiotracer ^{18}F FDG. This falls into the low risk category of the ARPANSA guidelines. This is outlined in the radiation assessment report (Appendix A). There is a small risk of perturbation in blood glucose levels following ^{18}F FDG administration. To minimize

this risk all participants will undergo blood glucose testing at screening and further testing prior to any administration if there are clinical concerns to ensure they are suitable to receive the radiotracer. There is a very small risk of a reaction to the infusion of the radiotracer ^{18}F FDG. This is outlined in section 8.4. A product information sheet for ^{18}F FDG will be provided to each participant (Appendix G.)

There is the risk of incidental abnormalities being identified on whole body PET/MRI imaging. These will be managed on an individual basis in consultation between the reporting radiologist, study doctor, principal investigator and participant.

Other risks, including those associated with IBSM inoculation and blood collection are as described in the MAIN study. EXPLORATORY STUDY involvement is not expected to alter these pre-existing risks.

<u>Potential Benefits</u>

There are no direct health benefits from participation in this EXPLORATORY STUDY.

Other potential benefits are as described in the MAIN study.

3. STUDY DESIGN

This is an EXPLORATORY STUDY comprising a population recruited from a single-center, IBSM model MAIN study. This EXPLORATORY STUDY is a pilot investigation to establish the role of ^{18}F FDG-PET/MRI in studying the pathophysiology of subpatent malaria. The population will consist of two healthy adults inoculated with *P. falciparum* from existing MAIN study population.

MAIN study refers to the parallel IBSM MAIN study QP15C20 SJ733

Participants will receive **two** whole body PET/MRI scans: the first within one week prior to MAIN study inoculation and the second one to two days prior to confinement with peak parasitaemia post BSP inoculation (estimated MAIN study InD 6 to 7). Blood samples will be collected at MAIN study timepoints.

Preparation:
At screening, participants will be consented for EXPLORATORY STUDY inclusion. At the time of consent the participant and study doctor will complete the MRI checklist (see Appendix B) and education regarding pre-imaging preparation. In the 24 hours prior to image collection, a low carbohydrate diet is to be followed, and strenuous exercise is to be avoided. These activities are to be recorded on the provided diet and activity sheet (see Appendix C).

On the day of imaging, participants are advised to wear warm clothing and arrive fasted for 6 hours prior (water is permitted, and good hydration encouraged). Participants will arrive to HIRF where the MRI checklist and diet and activity checklist will be reviewed. A peripheral intravenous cannula will be inserted for radiotracer administration. A bedside blood glucose measurement will be measured if there is

clinical suspicion of a blood glucose abnormality.

Whole body imaging will be performed on the Biograph mMR PET/MRI system after the intravenous infusion of the radiotracer ^{18}F FDG. Dynamic tracer uptake will be recorded over a 45-60 minute period from the abdomen for quantitative measurement of FDG uptake in the spleen. Participants will be offered a short break, followed by collection of static images of the whole body and brain, over an estimated 30 minute period.

Dedicated brain imaging will take place using the MAGNETOM Prisma 3T MRI system. This includes MP2RAGE, T2FLAIR and diffusion weighted MRI sequences of the brain for assessment of the effects of very subtle oedema and inflammatory responses. The estimated time of image acquisition is 45 minutes per patient. Depending on initial results, there may be some changes to MRI sequences to improve data collection. Participant activities or exposures will not be affected by any technical imaging sequence change.

Following image acquisition participants will be allowed food and drink and encouraged to drink water. Each HIRF visit is approximately 4 hours in total duration.

Image Interpretation:
Collected images will be reviewed and reported by specialist radiologists at HIRF/RBWH. Imaging metrics (FDG uptake and kinetic parameters) will be compared between baseline and follow up scans. Quantitative ^{18}F FDG uptake will be calculated using Patlak model analysis from dynamic uptake imaging, yielding a Ki value (^{18}F FDG influx constant.) A nominated region of interest will have quantitative ^{18}F FDG uptake calculated for each scan. Semi-quantitative ^{18}F FDG uptake will be calculated using SUVs measured during static uptake imaging. All regions of interest will have SUVs calculated.

Other data collection:
Other blood samples will be collected as per the MAIN study.

Evaluation of data:
All data will be presented descriptively. Pre and post inoculation quantitative imaging metrics will be compared with paired T-tests or Mann-Whitney U tests. Groups will be compared with unpaired T-tests with consideration of T-value adjustment for population size and Mann-Whitney U tests. Any groups identified based on imaging results will be described with respect to demographic, clinical and biochemical data collected as part of the MAIN study.

3.1 Study Flow Chart

	Screening Visit	*Within one week prior to MAIN study inoculation*	*MAIN study one to two days prior to confinement (~InD 6 to 7)*

Patient Information and Informed Consent	*X*		
Review of MRI checklist	*X*	*X*	*X*
Review of diet and activity sheet	*X*	*X*	*X*
Whole body PET/MRI		*X*	*X*
Dedicated brain MRI		*X*	*X*
Fasted blood glucose measurement	*X*	*X**	*X**

* If clinical concerns

4. PARTICIPANT ENROLLMENT AND WITHDRAWAL

4.1 Recruitment

Following receipt and signing the MAIN study consent forms, subjects will be fully informed of the nature of this optional EXPLORATORY STUDY, and the specific risks associated with this EXPLORATORY STUDY. A separate ‘Participation Information Sheet and Consent Form’ will be provided for this purpose (Appendix D).

The ‘Informed Consent’ will be signed and dated by the participants in the presence of an investigator. Subjects will also be given a copy of their signed ‘Informed Consent’. A copy of the HIRF site map (Appendix E), diet and activity sheet (Appendix C) and ^{18}F FDG Product Information (FDA information, see section 12. Appendix G) will be provided for participant reference.

4.2 Eligibility Criteria

4.2.1 Inclusion Criteria

In order to be eligible to participate in this study, an individual must meet all of the following criteria:

- Provide signed and dated informed consent form
- Able to lie supine and still for duration of image acquisition
- All other criteria as outlined in MAIN study protocol(s)

4.2.2 Exclusion Criteria

An individual who meets any of the following criteria will be excluded from participation in the study:

- Known allergic reactions to components of the study radiotracer ^{18}F FDG
- Fasted screening blood glucose elevated above the normal range (BSL >6.0mmol/L)
- Failure to meet/provide the standard MRI checklist requirements
- Claustrophobia precluding image acquisition
- Significant previous radiation exposure as defined (lifetime exposure):
  - Any fluoroscopic imaging (e.g. coronary angiography)
  - Any nuclear medicine imaging (e.g. myocardial perfusion scan)
  - Greater than one previous CT scan

   - *Note:* previous plain film X-Rays and mammography are acceptable
- All other criteria as outlined in MAIN study

4.3 Participant Withdrawal

Participants have the right to withdraw from the study at any time for any reason. The investigator also has the right to withdraw patients from the study in the event of any clinical adverse event (AE), laboratory abnormality, or other medical condition or situation occurs such that continued participation in the study would not be in the best interests of the participant OR the participant meets and exclusion criterion (either newly developed or not previous recognized) that precludes further study participation.

Following consent, data acquired up until withdrawal will be included in the EXPLORATORY STUDY. No further samples will be collected from the time of withdrawal notification.

4.4 Permanent Termination or Suspension of Exploratory Study

The principal investigator(s), Human Research Ethics Committee (HREC) and Regulatory Authorities independently reserve the right to discontinue the study at any time for safety or other reasons. This will be done in consultation with the MAIN study sponsor where practical. The MAIN study sponsor, in consultation with the investigators may request for suspension of the EXPLORATORY STUDY.

After such a decision, the investigator(s) will ensure that adequate consideration is given to the projection of the participants' interests. The investigator must review all participants as soon as practical and complete all required records.

5. STUDY INVESTIGATIONS

Whole body imaging will be performed using the Biograph mMR PET/MRI system, with concurrent PET and MRI acquisition. Dedicated Brain MRI sequences will be performed using the MAGNETOM Prisma 3T MRI system. This equipment is property of HIRF.

The standard radiotracer ^{18}F FDG will be used for PET imaging. This radiotracer has previously been approved by the Australian Register of Therapeutic Goods as a consumable for use in PET imaging (Austin Health) and attracts an MBS rebate for use in multiple conditions.

The radiotracer is to be purchased from the RBWH Nuclear Medicine Department, a TGA licensed manufacturer of ^{18}F FDG. The ^{18}F FDG is produced under the Good Manufacturing Practice (GMP) conditions in accordance with the British Pharmacopeia (see Appendix F.). The research will not alter the formulation of the product. The research will be using an identical product in an identical way in an identical formulation to that used for clinical diagnostic PET imaging. ^{18}F FDG is supplied as a clear/colourless or slightly yellow solution containing the radionuclide ^{18}F conjugated to the biologically active ligand glucose. It is administered as an

intravenous injection prior to imaging.

All radiotracer for use will be released as per existing RBWH Nuclear Medicine department practices. Dosing of the radiotracer is at a standard weight-based dosage. This is determined based on participant radiation exposure, not pharmacological effect. The effective dose of the metabolically active ligand (FDG) is considered a micro-dose.

Due to the unique nature of radiopharmaceuticals ^{18}F FDG is considered an investigational product by the TGA for purposes of this research project.

5.1 Radiotracer Dosing Regimen

- Prior to administration of ^{18}F FDG participants are to fast for roughly 6 hours.
- Prior to administration of ^{18}F FDG participants are to avoid strenuous exercise and adhere to a low carbohydrate diet for 24 hours.

An estimated dose of 4.5MBq per kg (based on screening weight) ^{18}F FDG is to be administered by infusion (minimum dose 90MBq, maximum dose 400MBq). This is the lowest possible dose to yield reliable data. The radioactive half-life of ^{18}F FDG is 110 minutes. Estimated radiation exposure is included in the radiation assessment report (see Appendix A).

- Following administration of ^{18}F FDG participants are encouraged to drink water to promote the renal excretion of radiotracer.

5.2 Modification of Radiotracer Administration for a Participant

Any participant experiencing an AE to any part of the investigation (participation in imaging process or infusion of radiotracer) will be individually evaluated. Where it is considered unsafe to repeat the process by the investigator(s) in discussion with the medical monitor, no further imaging will take place.

5.3 Participant Compliance

Participant non-compliance with preparation for image acquisition or non-compliant with instruction during imaging itself (e.g. excessive movement) will be recorded in the participant file as this may have an impact on the quality of data collected. This will include reference to the diet and exercise activity sheet assessed prior to imaging.

5.4 Radiotracer Manufacture, Handling and Accountability

The formulation, packaging and labeling of the standard radiotracer ^{18}F FDG is defined by licensed manufacturer. The ^{18}F FDG is stored at room temperature in accordance to the conditions set by the manufacturer. Handling of the radiopharmaceutical will follow the standard HIRF protocols for all injectable radiopharmaceuticals

The ^{18}F FDG will be ordered on an as needed basis. The ^{18}F FDG is supplied on the day of each study. The product expiry time is on the same day as supply due to the short ½ life of the radioactive element. There will be no excess product. The

administration of the GMP approved standard radiotracer ^{18}F FDG will be as per existing HIRF practices, including record keeping of ^{18}F FDG lot number, patient injected activity and time of administration using the Venstra system. Product accountabilty and quality control will be as per existing RBWH Nuclear Medicine Department practices under the Quality GMP Agreement for TGA licensing for the manufacture and supply of radiopharmaceuticals.

6. STUDY SCHEDULE

6.1 Screening

Participants consenting for participation in this EXPLORATORY STUDY must meet all inclusion and exclusion criteria for both MAIN and EXPLORATORY STUDY participation.

This includes EXPLORATORY STUDY specific screening for:

- Fasted blood glucose (exclude if >6.0mmol/L)
- Significant prior radiation exposure precluding participation (see exclusion criteria)
- MRI checklist completion, including evaluation for claustrophobia and ability to comfortably lie supine that may preclude participation
- Evaluation of patient understanding of preparation for imaging acquisition instructions: fast 6 hours prior; abstain from vigorous exercise and adherence to low carbohydrate diet 24 hours prior.

6.2 Enrollment/Baseline

Baseline fasted blood glucose and MRI checklist must be completed. Low carbohydrate diet options will be discussed.

6.3 Intermediate Visits

The first visit (within one week prior to MAIN study inoculation) will include whole body PET/MRI scanning.

6.4 Final Exploratory Study Visit

The second visit (MAIN study one to two days prior to confinement, InD 6 to 7) will include whole body PET/MRI scanning. This will also be the final study visit. Any AEs that require follow up will be reviewed at subsequent MAIN study visits.

6.5 Withdrawal Visit

A withdrawal visit coinciding with a scheduled MAIN study visit (or MAIN study withdrawal visit) will take place to review imaging results as required.

6.6 Unscheduled Visit

There are no unscheduled visits expected for this EXPLORATORY STUDY. In any case where an unscheduled visit is required, attempts will be made to ensure this occurs at a pre-existing MAIN study visit.

7. STUDY PROCEDURES AND EVALUATIONS

7.1 Study Procedures

Medical history, medication history, physical examination and biological specimen collection as per MAIN study.

EXPLORATORY STUDY specific procedures include:

Screening visit:

- Completion of MRI checklist at screening visit – consultation between participant and study doctor
- Recording of radiation exposure history at screening visit – consultation between participant and study doctor
  - radiology including plain films, CT scans, mammography, fluoroscopy and nuclear medicine (both diagnostic and interventional).
- Participant education regarding preparation for imaging
  - Low carbohydrate diet advice and information sheet
  - Discussion of physical activity limitation in pre-imaging period

Attendance for imaging:

- Insertion of peripheral intravenous cannula (as per MAIN study) for radiotracer administration
- Capillary blood glucose measurement if clinical concerns
- Whole body PET/MRI imaging
  - abstinence from physical activity for >24 hours prior recorded (or type of activity recorded for consideration during interpretation if participant noncompliant with instruction)
  - low carbohydrate diet for >24 hours prior and fasting for roughly 6 hours prior recorded
  - insertion of a peripheral intravenous cannula
  - review of MRI checklist
  - infusion of ^{18}F FDG via peripheral intravenous cannula
  - image acquisition – participant supine on scanner for duration of image collection
  - image review and reporting by specialist radiologist
  - ^{18}F FDG uptake quantification by Patlak model analysis by medical physicist, SUV semiquantification of remaining regions of interest
- Dedicated brain MRI imaging
  - image acquisition – participant supine on scanner for duration of image collection
  - image review and reporting by specialist radiologist

7.2 Laboratory Procedures/Evaluations

CLINICAL LABORATORY EVALUATIONS

MAIN study fasted blood glucose measurements will be used as part of the EXPLORATORY STUDY screening process.
Bedside capillary blood glucose measurement will take place prior to PET/MRI scanning if there are clinical concerns.
MAIN study baseline and serial safety blood tests (full blood count and biochemistry) will be used for comparison of imaging results.

SPECIAL ASSAYS OR PROCEDURES

Collection of blood samples for parasitaemia and parasite biomass measurements will take place as part of the MAIN study protocols.

8. ASSESSMENT OF SAFETY

8.1 Specification of Safety Parameters

As per MAIN study

8.2 Definition of an Adverse Event and of a Serious Adverse Event

ADVERSE EVENT

Any untoward medical occurrence in a patient or clinical investigation participant administered a pharmaceutical product and which does not necessarily have a causal relationship with this treatment. An AE can therefore be any unfavorable and unintended sign (including an abnormal laboratory finding), symptom, or disease temporally associated with the use of a product, whether or not related to the product.

SERIOUS ADVERSE EVENT

A serious AE is any untoward medical occurrence that, at any dose:

a) Results in death
b) Is life threatening (the term ‘life-threatening’ in the definition of ‘serious’ refers to an event in which the participant was at risk of death at the time of the event. If does not refer to an event, which hypothetically might have caused death if it were more severe.
c) Required hospitalization or prolongation of an existing hospitalization (hospitalization for elective treatment of a pre-existing condition that did not worsen from baseline is not considered an AE)
d) Results in disability/incapacity
e) Is a congenital abnormality/birth defect.

Use medical and scientific judgment when deciding whether reporting is appropriate in other situations, such as other important medical events that may not be immediately life-threatening or result in death or hospitalization, but may jeopardize the participant or may require medical or surgical intervention to prevent one of the outcomes listed in the above definition.

SUSPECTED UNEXPECTED SERIOUS ADVERSE REACTION (SUSAR)

A suspected unexpected serious adverse reaction is an AE that is determined to be related to the radiotracer and is both serious and unexpected. The term ‘unexpected’ means that nature or severity of the event is not consistent with the applicable product information (Appendix G.)

8.3 Documentation and classification of study specific adverse events

For regulatory purposes to use the standard radiotracer ^{18}F FDG in this EXPLORATORY STUDY all AEs deemed related to radiotracer administration will be documented in an EXPLORATORY STUDY AE log including diagnosis, severity, toxicity, date of onset and resolution and any action taken. Toxicity will be determined using existing CTCAE version 4.0 criteria, consistent with those

proposed for use in the MAIN studies. Severity will be determined based on the following scale:

- Mild: does not interfere with participant's usual function
- Moderate: interferes to some extent with participant's usual function
- Severe: interferes significantly with participant's usual function

All AEs will be recorded in the MAIN study participant file as per MAIN study processes. Where an AE is deemed a result of EXPLORATORY STUDY activities, this will be made clear in documentation and discussed with the MAIN study principal investigator prior to any appropriate escalation to MAIN study medical monitor and sponsor.

8.4 Relationship to Study Investigation

For the purposes of this study for an AE to be considered related to ^{18}F FDG it must occur within 5 half-lives, or 550 minutes of administration. For an AE to be considered related to other imaging procedures, it must occur during the procedure itself.

Any AEs deemed related to the above EXPLORATORY STUDY procedures will be documented in the EXPLORATORY STUDY AE log by one the Investigator or one of his representatives.

<u>EXPECTEDNESS OF SAEs</u>

There are no specific expected SAEs from participation in whole body PET/MRI or dedicated brain MRI imaging.

Incidental abnormalities detected on imaging will be discussed between the reporting radiologist, study doctor, principal investigator and participant. If required, further review will be arranged by referral to the general practitioner or appropriate specialist. Any incidental findings are expected to be asymptomatic and unpredictable, given the study population of otherwise well, healthy adult volunteers.

There are no commonly reported adverse reactions to radiopharmaceuticals and ^{18}F FDG in the literature (Appendix G). A multicenter review of over 80 000 administrations found no reported AEs [12]. A single case of a probable cutaneous adverse drug reaction has been reported in an individual with multiple existing drug allergies [13]. This did not preclude the patient from repeated dosing. A single case episode of possible anaphylaxis has been reported in the context of the administration of multiple other medications [14]. In both cases symptomatic treatment was effective. Based on the volume of ^{18}F FDG use in the clinical setting, and paucity of published adverse events, the risk of such events is considered to be extremely low.

8.5 Recording and Prompt Reporting of Events

In all cases of AEs, the reporting process used for the MAIN study from which the participant was recruited will be used. If MAIN study procedures are not available recording and reporting of AEs will be in accordance with QIMRCTSOP013 and QIMRCTSOP016.

Any AE deemed related to the administration of ^{18}F FDG or other imaging procedures will be discussed where possible to further clarify causality with the nominated Nuclear Medicine Independent Medical Advisor.

Any SAE deemed related to the administration of ^{18}F FDG or other imaging procedures will be reported to the Sponsor within 24 hours following discussion with the medical monitor and where possible with the nominated Nuclear Medicine Independent Medical Advisor. The TGA and any other relevant authorities will be notified by the Sponsor (or institutionally approved representative) within 72 hours as per existing TGA guidelines.

REPORTING OF PREGNANCY

The MAIN study from which EXPLORATORY STUDY participants will be recruited will only include males and women of non-childbearing potential.

8.6 Halting Rules

The investigator(s) reserve the right to halt the study at any point. There are no specific safety findings expected that may prompt suspension or termination of the EXPLORATORY STUDY. In a situation where the MAIN study is halted, the EXPLORATORY STUDY will also be halted as required.

9. QUALITY ASSURANCE

EXPLORATORY STUDY materials and quality assurance related to the imaging of participants and any involvement onsite at HIRF will take place as per existing HIRF/RBWH processes. Data quality will be considered and described in the presentation of results.

The MAIN study is a registered clinical trial employing an external study monitor responsible for quality assurance with respect to inoculum and participant safety (including pathology testing and clinical examination/investigations.)

10. STATISTICAL CONSIDERATIONS

10.1 Sample Size

This is a pilot investigation to assess the application of functional nuclear medicine in subpatent malaria. The population will comprise of two adults (male and female of non-childbearing potential) participants between 18 and 55 years of age, recruited from the IBSM model MAIN study population.

It is expected that this study will be hypothesis generating. There are no pre-existing PET or prospective MRI imaging trials in malaria published in the literature. Data from this pilot investigation may be used in future power calculations if required.

10.2 Statistical Analysis Plan

All data will be presented descriptively. Pre and post inoculation quantitative imaging metrics will be compared with paired T-tests or Mann-Whitney U tests (statistical

significance $p<0.05$). Imaging results may be described with respect to demographic, clinical and biochemical data collected as part of the MAIN study.

11. SOURCE DOCUMENTS AND ACCESS

Upon request, the investigator(s)/institution(s) will permit direct access to source data/ documents for trial-related monitoring, audits, HREC review, and regulatory inspection(s) by the Sponsor (or their appropriately qualified delegate) and Regulatory Authorities provided this is feasible from a technical standpoint. Direct access includes examination, analysis, verification and reproduction of records and reports that are important to the evaluation of the study.

12. ADMINISTRATIVE PROCEDURES

ETHICAL CONSIDERATIONS

The amount of blood to be sampled in the study is not considered to be excessive in healthy adult participants. This study will be carried our according to the Declaration of Helsinki, the NHMRC National Statement on Ethical Conduct in Human Research (2007) and the Notes for Guidance on Good Clinical Practice as adopted by the Australian Therapeutic Goods Administration (2000) (CPMP/CH/135/95) and the ICH GCP Guidelines.

ETHICAL REVIEW COMMITTEE

This Protocol will be submitted for approval to QIMR-B HREC, Clinical Trial Protocol Committee. This protocol will then be submitted for executive approval from RBWH HREC. Following this the HIRF Scientific Advisory Committee will review the Protocol and QIMR-B HREC assessment(s) in order to facilitate expedited Site-Specific Approval from the RBWH/Metro North HREC.

Written approvals will be obtained before volunteers are recruited and participants are enrolled. The Investigators will receive all the documentation needed for submitting the present Protocol to the HREC.

It is the responsibility of the Investigator to report study progress to the HREC as required or at intervals not greater than one year.

An earlier version of this protocol was submitted to the QIMR Clinical Trials Protocol Committee and was referred for review by the Human Scientific Sub-Committee on the grounds that the proposed study is not a clinical trial. Subsequent discussion with the TGA and study sites have established that the HREC review process described above is required to meet the regulatory requirements for research using the standard radiotracer ^{18}F FDG and between the nominated sites.

REGULATORY AUTHORITIES

This is an EXPLORATORY STUDY to run in parallel to an existing MAIN study clinical trial. The investigation uses the application of imaging using a standard radiotracer at standard dosages that is widely used in the clinical setting. This is administered as per existing local institution processes. Due to the unique nature of radiopharmaceuticals, facilities are able to extemporaneously produce and administer FDG onsite for their own purposes. The RBWH Nuclear Medicine department has TGA approval for manufacture of this radiotracer (Appendix F). This radiotracer is not ARTG listed for this facility, but is approved for GMP production and commercial sale from this manufacturer to other facilities within the state of

Queensland. Following discussion with the TGA and study sites, this EXPLORATORY STUDY has a CTN registration for the **regulatory purpose** of accessing the standard radiotracer ^{18}F FDG in a manner that ensures prospective record keeping and transparent usage. This includes processes for AE reporting as documented below.

An independent nuclear medicine medical advisor has been nominated to ensure participant safety, to provide advice and ongoing review of any SAEs that may be related to this EXPLORATORY STUDY activities. No DSMB has been nominated given the low risk nature of this small population EXPLORATORY STUDY. Participants will receive a copy of a current FDA template for ^{18}F FDG product information (Appendix G), as no generic equivalent exists in Australia.

INFORMED CONSENT

Before recruitment and enrolment into the study, each prospective participant must be given a full explanation of the nature and purpose of the study, and a copy of the Participant Information Sheet and Consent Form to review. This form complies with the consent form requirements outlined in the TGA guidelines. Once the essential study information has been provided, and the Investigator is assured that each individual volunteer understands the implications of participating in the study, the participants will be asked to give consent to participate in the study by signing the informed consent form. The consent form shall be signed and dated by the appropriate parties. A notation that written informed consent has been obtained will be made on the participants' MAIN study file. The completed consent forms will be retained by the Investigator and a copy of these will be provided by the Investigator to the participant.

PARTICIPANT RE-IMBURSEMENT

Participants who are found eligible and complete the study will be compensated $160 for their time. This is in addition to MAIN study compensation.

EMERGENCY CONTACT WITH INVESTIGATORS

All participants will be provided with a Participant Information Sheet and Informed Consent Form with contact details of whom to contact in the case of an emergency related to the EXPLORATORY STUDY.

Participants will also be provided with a contact card as per the MAIN study.

NOTIFICATION OF PRIMARY CARE PHYSICIAN

Where it is deemed appropriate by the investigator(s), study doctor and/or reporting radiologist a letter will be sent to the physician stating the nature of the EXPLORATORY STUDY including any adverse events or incidental findings. A copy shall be retained by the study site as part of the MAIN study participant file.

PROTOCOL DEVIATIONS

All protocol deviations must be reported to the principal investigator. Protocol deviations will be assessed for significance by the principal investigator. Those deviations deemed to have a potential impact on the integrity of the study results, patient safety or the ethical acceptability of the trial will be reported to the HREC prior to completion of participant enrollment. Where deviations to the protocol identify issues for protocol review, the protocol may be amended as above.

RETENTION OF OTHER STUDY SPECIFIC SAMPLES

Electronic imaging data will be retained as per HIRF guidelines. No other samples will be collected.

INSURANCE AND INDEMNITY

This EXPLORATORY STUDY is an observational study to run parallel with existing MAIN study clinical trials. There are no specific expected SAEs from participation requiring indemnity, however in the event of an AE that is deemed related to EXPLORATORY STUDY participation (following Investigator and medical monitor review) the sponsor holds No-Fault Compensation for Clinical Trials insurance policy for this study. The site HIRF is part of the RBWH campus.

The MAIN study holds a No-Fault Compensation for Clinical Trials insurance policy.

FINANCIAL DISCLOSURE AND CONFLICT OF INTEREST

Nil conflicts of interest identified or disclosed.

13. DATA HANDLING, RECORD KEEPING AND PUBLICATION POLICY

DATA CONFIDENTIALITY

Only the investigators, designated staff and designated MAIN study staff will have access to information that identifies a study participant.
All MAIN study participants will be assigned a unique identifier.
Participant identifiers in this EXPLORATORY STUDY will be consistent with those used in corresponding MAIN studies.

DATA CAPTURE METHODS

All imaging data will be collected in accordance with site-specific HIRF protocols.

TYPES OF DATA

Imaging data, reports and quantitative imaging metrics will be electronically stored in accordance with HIRF guidelines.

RECORD KEEPING

All study related documents and records are to be retained for a minimum of fifteen years after MAIN study completion. Written agreement from the Sponsor must precede destruction of the same. In cases where HIRF guidelines preclude the storage of source imaging data, data will be transferred to the sponsor for storage.

PUBLICATIONS POLICY

Publication and reporting of results and outcomes of this trial will be accurate and honest, undertaken with integrity and transparency and in accordance with QIMR BERGHOFER's Policy on Criteria for Authorship. Publication of results will be subjected to fair peer-review. Authorship will be given to all persons providing significant input into the conception, design, execution or reporting of the research according to QIMR BERGHOFER Policy on the Criteria for Authorship.

14. LITERATURE REFERENCES

1. Khoury, D.S., et al., *Effect of mature blood-stage Plasmodium parasite sequestration on pathogen biomass in mathematical and in vivo models of malaria.* Infect Immun, 2014. **82**(1): p. 212-20.
2. Dondorp, A.M., et al., *Estimation of the total parasite biomass in acute falciparum malaria from plasma PfHRP2.* PLoS Med, 2005. **2**(8): p. e204.
3. Barber, B.E., et al., *Parasite biomass-related inflammation, endothelial activation, microvascular dysfunction and disease severity in vivax malaria.* PLoS Pathog, 2015. **11**(1): p. e1004558.
4. Lopes, S.C., et al., *Paucity of Plasmodium vivax mature schizonts in peripheral blood is associated with their increased cytoadhesive potential.* J Infect Dis, 2014. **209**(9): p. 1403-7.
5. Anstey, N.M., et al., *Lung injury in vivax malaria: pathophysiological evidence for pulmonary vascular sequestration and posttreatment alveolar-capillary inflammation.* J Infect Dis, 2007. **195**(4): p. 589-96.
6. Cromer, D., et al., *Where have all the parasites gone? Modelling early malaria parasite sequestration dynamics.* PLoS One, 2013. **8**(2): p. e55961.
7. Franke-Fayard, B., et al., *Sequestration and tissue accumulation of human malaria parasites: can we learn anything from rodent models of malaria?* PLoS Pathog, 2010. **6**(9): p. e1001032.
8. Lacerda, M.V., et al., *Postmortem characterization of patients with clinical diagnosis of Plasmodium vivax malaria: to what extent does this parasite kill?* Clin Infect Dis, 2012. **55**(8): p. e67-74.
9. Roth, E., Jr., *Plasmodium falciparum carbohydrate metabolism: a connection between host cell and parasite.* Blood Cells, 1990. **16**(2-3): p. 453-60; discussion 461-6.
10. Yeo, T.W., et al., *Impaired skeletal muscle microvascular function and increased skeletal muscle oxygen consumption in severe falciparum malaria.* J Infect Dis, 2013. **207**(3): p. 528-36.
11. Laothamatas, J., et al., *Transient lesion in the splenium of the corpus callosum in acute uncomplicated falciparum malaria.* Am J Trop Med Hyg, 2014. **90**(6): p. 1117-23.
12. Silberstein, E.B., *Prevalence of adverse reactions to positron emitting radiopharmaceuticals in nuclear medicine. Pharmacopeia Committee of the Society of Nuclear Medicine.* J Nucl Med, 1998. **39**(12): p. 2190-2.
13. Codreanu, I., et al., *Fluorodeoxyglucose-induced allergic reaction: a case report.* J Oncol Pharm Pract, 2013. **19**(1): p. 86-8.
14. Lee, D.Y., et al., *An unusual case of anaphylaxis after fluorine-18-labeled fluorodeoxyglucose injection.* Nucl Med Mol Imaging, 2013. **47**(3): p. 201-4.

- World Medical Association Declaration of Helsinki – Ethical Principles for Medical Research Involving Human Subjects
- NH&MRC National Statement on Ethical Conduct in Human Research (2007).
- Notes for Guidance on Good Clinical Practice – Annotated with TGA Comments (CPMP/ICH/135/95), as adopted by the Australian Therapeutic Goods Administration (July 2000).
- QIMR BERGHOFER Standard Operating Procedures

Exploratory Study Protocol

Advanced medical imaging in subpatent malaria: a second pilot study

Protocol No: P2261
Version: 1.0
Date: 30September2016

For addition to parallel malaria challenge studies, as outlined in specific challenge study protocol (exploratory substudy)

ACTRN12616001238460 linked to IBSM study ACTRN12616000174482

PROTOCOL SYNOPSIS

Full Title: Advanced medical imaging in subpatent malaria: a pilot study

Short Title: NA

Objectives:

Primary:

- To investigate the use of ^{18}F FDG-PET/MRI to estimate the biodistribution of *Plasmodium* species in human volunteer induced blood stage malaria studies.
- To investigate the use of ^{18}F FDG-PET/MRI to estimate the biomass of *Plasmodium* species*.*

Secondary:

- To describe the relative burden of organ-specific tissue sequestration in *Plasmodium* species*.*
- To describe the impact of early malaria infection on glucose metabolism.
- To describe the MRI brain findings of early malaria infection in a prospective participant cohort.

Population: Eight adult (male and non-lactating, non-pregnant female) participants between 18 and 55 years of age, recruited from induced blood stage malaria (IBSM) model MAIN study populations under QIMR-Berghofer sponsorship.

Phase: exploratory

Number of Sites:

- Q-Pharm Pty Limited, Herston, QLD, Australia
- Herston Imaging Research Facility (HIRF), Herston, QLD, Australia

Description of Investigation:

Participants will receive a whole body PET/MRI scan and dedicated MRI scan within roughly one week prior to inoculation and on one to two days prior to confinement with peak parasitaemia post BSP inoculation (estimated InD 6 to 7 for *P. falciparum* and InD 8 to 9 for *P. vivax*). Preparation for PET/MRI imaging involves fasting for 6 hours prior, abstaining from excessive physical exertion and following a low carbohydrate diet 24 hours prior (recorded on a diet and activity sheet). PET/MRI scans will be performed on the Biograph mMR PET/MRI system after the intravenous infusion of the standard radiotracer 2-(^{18}F) fluor-deoxy-D-glucose(FDG) (^{18}F FDG) and dedicated MRI scans on the MAGNETOM Prisma 3T MRI system. Collected images will be reviewed and reported by experienced radiologists specialising in MRI and nuclear medicine reporting. Quantification of ^{18}F FDG uptake measurements will be established for intra-individual scans using Patlak model analysis and semi quantitative SUV measurement with reference to an ^{18}F FDG external control.

Study Duration: October 2016 – June 2017

Participant Participation Duration: Estimated 8-14 days

Estimated Time to Complete Enrollment: August 2016 – March 2017

1. KEY ROLES AND CONTACT INFORMATION 9
1.1 Study Location(s) 10
1.2 Study Management 10
2. INTRODUCTION: BACKGROUND AND SCIENTIFIC RATIONALE 11
2.1 Background Information 11
2.2 Study Objectives 12
3. STUDY DESIGN 14
3.1 Study Flow Chart 16
4. PARTICIPANT ENROLLMENT AND WITHDRAWAL 16
4.1 Recruitment 16
4.2 Eligibility Criteria 16
4.3 Participant Withdrawal 17
4.4 Permanent Termination or Suspension of Exploratory Study 17
5. STUDY INVESTIGATIONS 17
5.1 Radiotracer Dosing Regimen 18
5.2 Modification of Radiotracer Administration for a Participant 18
5.3 Participant Compliance 18
5.4 Radiotracer Manufacture, Handling and Accountability 19
6. STUDY SCHEDULE 19
6.1 Screening 19
6.2 Enrollment/Baseline 19
6.3 Intermediate Visits 19
6.4 Final Exploratory Study Visit 19
6.5 Withdrawal Visit 20
6.6 Unscheduled Visit 20
7. STUDY PROCEDURES AND EVALUATIONS 20
7.1 Study Procedures 20
7.2 Laboratory Procedures/Evaluations 21
8. ASSESSMENT OF SAFETY 21
8.1 Specification of Safety Parameters 21
8.2 Definition of an Adverse Event and of a Serious Adverse Event 21
8.3 Documentation and classification of study specific adverse events 22
8.4 Relationship to Study Investigation 22
8.5 Recording and Prompt Reporting of Events 23
8.6 Halting Rules 23
9. QUALITY ASSURANCE 23
10. STATISTICAL CONSIDERATIONS 24
10.1 Sample Size 24

10.2 Statistical Analysis Plan 24
11. SOURCE DOCUMENTS AND ACCESS 24
12. ADMINISTRATIVE PROCEDURES 24
13. DATA HANDLING, RECORD KEEPING AND PUBLICATION POLICY 26
14. LITERATURE REFERENCES 27
15. APPENDICES 29
15.1 Appendix A: Radiation assessment Report 29
15.2 Appendix B: MRI checklist 30
15.3 Appendix C: Diet and Activity Sheet 31
15.4 Appendix D: Participant Information Sheet/Consent Form 32
15.5 Appendix E: HIRF site map 33
15.6 Appendix F: Licence to manufacture therapeutic goods 33
15.7 Appendix G: Product information 33

2. INTRODUCTION: BACKGROUND AND SCIENTIFIC RATIONALE

2.1 Background Information

Morbidity and mortality from malaria infection remains significant despite anti-parasitic treatments. Understanding the pathophysiology of disease may aid in the development of adjunctive therapies to improve survival in severe cases. Parasite sequestration in tissue microvasculature permits evasion of the reticulo-endothelial system and increased biomass [1]. Organ specific sequestration contributes to end organ dysfunction in severe malaria syndromes. Owing to the inaccessibility of sequestered parasites, estimations of total biomass and sites of sequestration have historically relied on biochemical markers [2-5], animal models [1, 6, 7] and post mortem studies [8]. Determination of the biodistribution of malaria with functional nuclear medicine imaging may allow for more direct study of sequestration in human models. This may aid developing a better understanding of the organ dysfunction experienced in severe cases.

Nuclear medicine imaging techniques, particular hybrid PET/CT and PET/MRI have a central role primarily in oncology for assessing the biodistribution and activity of malignancy. The ability of these techniques to detect and locate biological and biochemical changes have more recently been applied to other medical fields including Infectious Diseases, though there are no studies in malaria.

Other advanced imaging techniques including dedicated MRI imaging have been successfully applied to examine the pathophysiology of malaria, predominately in patients presenting with severe disease. There are few studies in early or uncomplicated infection, and none of these studies have been prospective. As a result there remains a paucity of imaging data in the early stages of infection.

This EXPLORATORY STUDY has been designed as a prospective pilot investigation to establish the role of ^{18}F FDG-PET/MRI and advanced dedicated MRI imaging techniques in studying the pathophysiology of subpatent malaria following low dose *Plasmodium* exposure in healthy adults. Collection of baseline and post inoculation

PET/MRI imaging will provide information about changes in host/parasite glucose metabolism that may be used to estimate parasite biomass and biodistribution. Glucose uptake is increased up to 100-fold in parasitised erythrocytes [9], suggesting that ^{18}F FDG may be a viable radiotracer to help evaluate the disease.

Whole body PET/MRI is a functional imaging modality that provides detailed soft tissue anatomical information with lower ionizing radiation exposure compared to equivalent PET/CT. The radiotracer ^{18}F FDG is a safe and well-validated biomimetic for demonstrating glucose uptake. Imaging with ^{18}F FDG-PET/MRI is an ideal model for exploring nuclear medicine functional imaging in a healthy human volunteer population.

Biochemical estimates of parasite biomass and parasitaemia will be compared to quantified radiotracer uptake measurements. Comparison of interval changes in host/parasite glucose metabolism will add to our understanding of the metabolic changes associated with disease [10]. The feasibility of ^{18}F FDG-PET/MRI may contribute to developing a further role for nuclear medicine imaging in malaria.

Dedicated MRI imaging can demonstrate inflammation other subtle metabolic changes, including changes in vascular function. Comparison of interval changes in dedicated MRI imaging will add to our understanding of early disease progression and permit comparison with the existing literature in more advanced disease.

Successful application of advanced medical imaging would provide a greater understanding of parasite sequestration dynamics, which may aid in disease modeling and development of treatments to prevent end organ damage in severe disease.

2.2 Study Objectives

Primary:

- To investigate the use of ^{18}F FDG-PET/MRI to estimate the biodistribution of *Plasmodium* species in human volunteer induced blood stage malaria studies.
- To investigate the use of ^{18}F FDG-PET/MRI to estimate the biomass of *Plasmodium* species.
- To investigate the use of advanced dedicated MRI techniques to early *Plasmodium* infection.

Secondary:

- To describe the relative burden of organ-specific tissue sequestration in *Plasmodium* infection.
- To describe the impact of early malaria infection on glucose metabolism.
- To describe the MRI brain findings of early malaria infection in a prospective participant cohort.

i. Research Question

The present EXPLORATORY STUDY has been designed to establish the role of ^{18}F FDG-PET/MRI and advanced dedicated MRI techniques in studying the

pathophysiology of subpatent malaria following low dose *Plasmodium* exposure in healthy adults.

ii. Study Outcome Measures

<u>Primary</u>

The primary objective of the study is to assess the application of whole body ^{18}F FDG-PET/MRI in describing the parasite biodistribution and biomass in subpatent malaria. This is a hypothesis generating pilot investigation expected to have predominately descriptive outcomes.

The primary outcome measures will be the quantified and semi-quantified ^{18}F FDG uptake values from specific regions of interest. Regions of interest for quantification of uptake will be spleen, bone marrow (lumbar spine and/or pelvis), muscle bulk (quadriceps) and brain. Post inoculation measurements will be compared to baseline pre inoculation uptake values. Biochemical markers of parasitaemia and total biomass from MAIN study data will be compared to ^{8}F FDG uptake values.

Any other regions of interested (as deemed by the investigators) identified after imaging will have semi-quantified ^{18}F FDG uptake values calculated for further comparison.

The other primary objective seeks to apply advanced MRI techniques to early *Plasmodium* infection. This will generate descriptive outcomes and inform future MRI sequence selection to optimize imaging in malaria.

<u>Secondary</u>

The relative burden of organ specific sequestration will be determined by comparing ^{18}F FDG uptake values for each region of interest.

The impact of early malaria infection on glucose metabolism will be evaluated in assessing each of the above outcomes.

The MRI findings of early malaria will be presented in a descriptive manner, outlining any interval changes in high-resolution brain MRI for this prospective cohort.

iii. Study Rationale

This EXPLORATORY STUDY has been designed to establish the role of ^{18}F FDG-PET/MRI and advanced MRI techniques in studying the pathophysiology of subpatent malaria. The selection of this radiotracer and imaging modality offers a well-validated model for exploring nuclear medicine functional imaging of malaria infection in a healthy human volunteer population.

Hypotheses

- Whole body ^{18}F FDG-PET/MRI is a technically feasible imaging modality for the study of subpatent malaria infection.

- Quantitative and semi-quantitative uptake of ^{18}F FDG is proportional to estimated parasite biomass and parasitaemia.
- Subtle changes in the vasculature of the brain are present and identifiable on MRI in subpatent malaria infection.

iv. Potential Risks and Benefits

Potential Risks

There is a small risk associated with radiation exposure from the radiotracer ^{18}F FDG. This falls into the low risk category of the ARPANSA guidelines. This is outlined in the radiation assessment report (Appendix A). There is a small risk of perturbation in blood glucose levels following ^{18}F FDG administration. To minimize this risk all participants will undergo blood glucose testing at screening and further testing prior to any administration if there are clinical concerns to ensure they are suitable to receive the radiotracer. There is a very small risk of a reaction to the infusion of the radiotracer ^{18}F FDG. This is outlined in section 8.4. A product information sheet for ^{18}F FDG will be provided to each participant (Appendix G.)

There is the risk of incidental abnormalities being identified on whole body PET/MRI imaging. These will be managed on an individual basis in consultation between the reporting radiologist, study doctor, principal investigator and participant.

Other risks, including those associated with IBSM inoculation and blood collection are as described in the MAIN study. EXPLORATORY STUDY involvement is not expected to alter these pre-existing risks.

Potential Benefits

There are no direct health benefits from participation in this EXPLORATORY STUDY.

Other potential benefits are as described in the MAIN study.

3. STUDY DESIGN

This is an EXPLORATORY STUDY comprising a population recruited from single-center, IBSM model MAIN studies. The population will consist of up to eight healthy adults inoculated with *Plasmodium* species parasite from existing MAIN study population.

MAIN study refers to the parallel IBSM MAIN study from which the participant is drawn from.

Participants will receive **two** scans: the first within one week prior to MAIN study inoculation and the second one to two days prior to confinement with peak parasitaemia post BSP inoculation (estimated InD 6 to 7 for *P. falciparum* and InD 8 to 9 for *P. vivax*). Blood samples will be collected at MAIN study timepoints, with no

additional collections planned for the EXPLORATORY STUDY.

Preparation:
At screening, participants will be consented for EXPLORATORY STUDY inclusion. At the time of consent the participant and study doctor will complete the MRI checklist (see Appendix B) and education regarding pre-imaging preparation. In the 24 hours prior to PET/MRI image collection, a low carbohydrate diet is to be followed, and strenuous exercise is to be avoided. These activities are to be recorded on the provided diet and activity sheet (see Appendix C).

On the day of PET/MRI imaging, participants are advised to wear warm clothing and arrive fasted for 6 hours prior (water is permitted, and good hydration encouraged). Participants will arrive to HIRF where the MRI checklist and diet and activity checklist will be reviewed. A peripheral intravenous cannula will be inserted for radiotracer administration. A bedside blood glucose measurement will be measured if there is clinical suspicion of a blood glucose abnormality.

Whole body PET/MRI imaging will be performed on the Biograph mMR PET/MRI system after the intravenous infusion of the radiotracer ^{18}F FDG. Dynamic tracer uptake will be recorded over a 45-60 minute period from the abdomen for quantitative measurement of FDG uptake in the spleen. Participants will be offered a short break, followed by collection of static images of the whole body and brain, over an estimated 30 minute period.

Dedicated brain imaging will take place using the MAGNETOM Prisma 3T MRI system. This includes MP2RAGE, T2FLAIR and diffusion weighted MRI sequences of the brain for assessment of the effects of very subtle oedema and inflammatory responses. The estimated time of image acquisition is 45 minutes per patient. Depending on initial results, there may be changes to MRI sequences to improve data collection. Participant activities or exposures will not be affected by any technical imaging sequence change.

Following image acquisition participants will be allowed food and drink and encouraged to drink water. A light meal will be provided prior to exit from HIRF. Each HIRF visit is approximately 4 hours in total duration.

Image Interpretation:
Collected images will be reviewed and reported by specialist radiologists at HIRF/RBWH. Imaging metrics (FDG uptake and kinetic parameters) will be compared between baseline and follow up scans. Quantitative ^{18}F FDG uptake will be calculated using Patlak model analysis from dynamic uptake imaging, yielding a Ki value (^{18}F FDG influx constant.) A nominated region of interest will have quantitative ^{18}F FDG uptake calculated for each scan. Semi-quantitative ^{18}F FDG uptake will be calculated using SUVs measured during static uptake imaging. All regions of interest will have SUVs calculated.

Other data collection:
Other blood samples will be collected as per the MAIN study.

Evaluation of data:

All data will be presented descriptively. Pre and post inoculation quantitative imaging metrics will be compared with paired T-tests or Mann-Whitney U tests. *Plasmodium spp.* groups will be compared with unpaired T-tests with consideration of T-value adjustment for population size and Mann-Whitney U tests. Any groups identified based on imaging results will be described with respect to demographic, clinical and biochemical data collected as part of the MAIN study.

3.1 Study Flow Chart

	Screening Visit	*Within one week prior to MAIN study inoculation*	*MAIN study one to two days prior to confinement*
Patient Information and Informed Consent	*X*		
Review of MRI checklist	*X*	*X*	*X*
Review of diet and activity sheet	*X*	*X*	*X*
Whole body PET/MRI		*X*	*X*
Dedicated brain MRI		*X*	*X*
Fasted blood glucose measurement	*X*	*X**	*X**

*If clinical concerns

4. PARTICIPANT ENROLLMENT AND WITHDRAWAL

4.1 Recruitment

Following receipt and signing the MAIN study consent forms, subjects will be fully informed of the nature of this optional EXPLORATORY STUDY, and the specific risks associated with this EXPLORATORY STUDY. A separate ‘Participation Information Sheet and Consent Form’ will be provided for this purpose (Appendix D).

The ‘Informed Consent’ will be signed and dated by the participants in the presence of an investigator. Subjects will also be given a copy of their signed ‘Informed Consent’. A copy of the HIRF site map (Appendix E), diet and activity sheet (Appendix C) and ^{18}F FDG Product Information (FDA information, see section 12. Appendix G) will be provided for participant reference.

4.2 Eligibility Criteria

4.2.1 Inclusion Criteria

In order to be eligible to participate in this study, an individual must meet all of the following criteria:

- Provide signed and dated informed consent form
- Able to lie supine and still for duration of image acquisition
- All other criteria as outlined in MAIN study protocol(s)

4.2.2 Exclusion Criteria

An individual who meets any of the following criteria will be excluded from participation in the study:

- Known allergic reactions to components of the study radiotracer ^{18}F FDG
- Fasted screening blood glucose elevated above the normal range (BSL >6.0mmol/L)
- Failure to meet/provide the standard MRI checklist requirements
- Claustrophobia precluding image acquisition
- Significant previous radiation exposure as defined (lifetime exposure):
    - Any fluoroscopic imaging (e.g. coronary angiography)
    - Any nuclear medicine imaging (e.g. myocardial perfusion scan)
    - Greater than one previous CT scan
    - *Note:* previous plain film X-Rays and mammography are acceptable
- All other criteria as outlined in MAIN study

4.3 Participant Withdrawal

Participants have the right to withdraw from the study at any time for any reason. The investigator also has the right to withdraw patients from the study in the event of any clinical adverse event (AE), laboratory abnormality, or other medical condition or situation occurs such that continued participation in the study would not be in the best interests of the participant OR the participant meets and exclusion criterion (either newly developed or not previous recognized) that precludes further study participation.

Following consent, data acquired up until withdrawal will be included in the EXPLORATORY STUDY. No further samples will be collected from the time of withdrawal notification.

4.4 Permanent Termination or Suspension of Exploratory Study

The principal investigator(s), Human Research Ethics Committee (HREC) and Regulatory Authorities independently reserve the right to discontinue the study at any time for safety or other reasons. This will be done in consultation with the MAIN study sponsor where practical. The MAIN study sponsor, in consultation with the investigators may request for suspension of the EXPLORATORY STUDY.

After such a decision, the investigator(s) will ensure that adequate consideration is given to the projection of the participants’ interests. The investigator must review all participants as soon as practical and complete all required records.

5. STUDY INVESTIGATIONS

Whole body imaging will be performed using the Biograph mMR PET/MRI system, with concurrent PET and MRI acquisition. Dedicated Brain MRI sequences will be performed using the MAGNETOM Prisma 3T MRI system. This equipment is property of HIRF.

The standard radiotracer ^{18}F FDG will be used for PET imaging. This radiotracer has previously been approved by the Australian Register of Therapeutic Goods as a consumable for use in PET imaging (Austin Health) and attracts an MBS rebate for use in multiple conditions.)

The radiotracer is to be purchased from the RBWH Nuclear Medicine Department, a TGA licensed manufacturer of ^{18}F FDG. The ^{18}F FDG is produced under the Good Manufacturing Practice (GMP) conditions (see Appendix F) in accordance with the British Pharmacopeia. The research will not alter the formulation of the product. The research will be using an identical product in an identical way in an identical formulation to that used for clinical diagnostic PET imaging. ^{18}F FDG is supplied as a clear/colourless or slightly yellow solution containing the radionuclide ^{18}F conjugated to the biologically active ligand glucose. It is administered as an intravenous injection prior to imaging.

All radiotracer for use will be released as per existing RBWH Nuclear Medicine department practices. Dosing of the radiotracer is at a standard weight-based dosage. This is determined based on participant radiation exposure, not pharmacological effect. The effective dose of the metabolically active ligand (FDG) is considered a micro-dose.

Due to the unique nature of radiopharmaceuticals ^{18}F FDG is considered an investigational product by the TGA for purposes of this research project.

5.1 Radiotracer Dosing Regimen

- Prior to administration of ^{18}F FDG participants are to fast for roughly 6 hours.
- Prior to administration of ^{18}F FDG participants are to avoid strenuous exercise and adhere to a low carbohydrate diet for 24 hours.

An estimated dose of 4.5MBq per kg (based on screening weight) ^{18}F FDG is to be administered by infusion (minimum dose 90MBq, maximum dose 400MBq). This is the lowest possible dose to yield reliable data. The radioactive half-life of ^{18}F FDG is 110 minutes. Estimated radiation exposure is included in the radiation assessment report (see Appendix A).

- Following administration of ^{18}F FDG participants are encouraged to drink water to promote the renal excretion of radiotracer.

5.2 Modification of Radiotracer Administration for a Participant

Any participant experiencing an AE to any part of the investigation (participation in imaging process or infusion of radiotracer) will be individually evaluated. Where it is considered unsafe to repeat the process by the investigator(s) in discussion with the Nuclear Medicine Independent Medical Advisor, no further imaging will take place.

5.3 Participant Compliance

Participant non-compliance with preparation for image acquisition or non-compliant with instruction during imaging itself (e.g. excessive movement) will be recorded in

the participant file as this may have an impact on the quality of data collected. This will include reference to the diet and exercise activity sheet assessed prior to imaging.

5.4 Radiotracer Manufacture, Handling and Accountability

The formulation, packaging and labeling of the standard radiotracer ^{18}F FDG is defined by licensed manufacturer. The ^{18}F FDG is stored at room temperature in accordance to the conditions set by the manufacturer. Handling of the radiopharmaceutical will follow the standard HIRF protocols for all injectable radiopharmaceuticals

The ^{18}F FDG will be ordered on an as needed basis. The ^{18}F FDG is supplied on the day of each study. The product expiry time is on the same day as supply due to the short half life of the radioactive element. There will be no excess product. The administration of the GMP approved standard radiotracer ^{18}F FDG will be as per existing HIRF practices, including record keeping of ^{18}F FDG lot number, patient injected activity and time of administration using the Venstra system. Product accountabilty and quality control will be as per existing RBWH Nuclear Medicine Department practices under the Quality GMP Agreement for TGA licensing for the manufacture and supply of radiopharmaceuticals.

6. STUDY SCHEDULE

6.1 Screening

Participants consenting for participation in this EXPLORATORY STUDY must meet all inclusion and exclusion criteria for both MAIN and EXPLORATORY STUDY participation.

This includes EXPLORATORY STUDY specific screening for:

- Fasted blood glucose (exclude if >6.0mmol/L)
- Significant prior radiation exposure precluding participation (see exclusion criteria)
- MRI checklist completion, including evaluation for claustrophobia and ability to comfortably lie supine that may preclude participation
- Evaluation of patient understanding of preparation for imaging acquisition instructions: fast 6 hours prior; abstain from vigorous exercise and adherence to low carbohydrate diet 24 hours prior.

6.2 Enrollment/Baseline

Baseline fasted blood glucose and MRI checklist must be completed. Low carbohydrate diet options will be discussed.

6.3 Intermediate Visits

The first visit (within one week prior to MAIN study inoculation) will include advanced medical imaging.

6.4 Final Exploratory Study Visit

The second visit (MAIN study one to two days prior to confinement) will include advanced medical imaging. This will also be the final study visit. Any AEs that require follow up will be reviewed at subsequent MAIN study visits.

6.5 Withdrawal Visit

A withdrawal visit coinciding with a scheduled MAIN study visit (or MAIN study withdrawal visit) will take place to review imaging results as required.

6.6 Unscheduled Visit

There are no unscheduled visits expected for this EXPLORATORY STUDY. In any case where an unscheduled visit is required, attempts will be made to ensure this occurs at a pre-existing MAIN study visit.

7. STUDY PROCEDURES AND EVALUATIONS

7.1 Study Procedures

Medical history, medication history, physical examination and biological specimen collection as per MAIN study.

EXPLORATORY STUDY specific procedures include:

Screening visit:

- Completion of MRI checklist at screening visit – consultation between participant and study doctor
- Recording of radiation exposure history at screening visit – consultation between participant and study doctor
  - radiology including plain films, CT scans, mammography, fluoroscopy and nuclear medicine (both diagnostic and interventional).
- Participant education regarding preparation for PET/MRI imaging
  - Low carbohydrate diet advice and information sheet
  - Discussion of physical activity limitation in pre-imaging period

Attendance for imaging:

- Insertion of peripheral intravenous cannula (as per MAIN study) for blood collection and radiotracer administration
- Capillary blood glucose measurement if clinical concerns
- Urine pregnancy testing for female participants of child bearing potential if not performed as part of MAIN study protocol within 24 hours prior.
- Whole body PET/MRI imaging
  - abstinence from physical activity for >24 hours prior recorded (or type of activity recorded for consideration during interpretation if participant noncompliant with instruction)
  - low carbohydrate diet for >24 hours prior and fasting for roughly 6 hours prior recorded
  - insertion of a peripheral intravenous cannula
  - review of MRI checklist
  - infusion of ^{18}F FDG via peripheral intravenous cannula
  - image acquisition – participant supine on scanner for duration of image collection
  - image review and reporting by specialist radiologist
  - ^{18}F FDG uptake quantification by Patlak model analysis by medical physicist, SUV semi-quantification of remaining regions of interest
- Dedicated MRI imaging
  - image acquisition – participant supine on scanner for duration of image collection

- image review and reporting by specialist radiologist

7.2 Laboratory Procedures/Evaluations

CLINICAL LABORATORY EVALUATIONS

MAIN study fasted blood glucose measurements will be used as part of the EXPLORATORY STUDY screening process.
Bedside capillary blood glucose measurement will take place prior to PET/MRI scanning if there are clinical concerns.
Urine point of care pregnancy testing will be performed at either of QPharm or HIRF within 24 hours prior to PET/MRI imaging.
MAIN study baseline and serial safety blood tests (full blood count and biochemistry) will be used for comparison of imaging results.

SPECIAL ASSAYS OR PROCEDURES

Collection of blood samples for parasitaemia and parasite biomass measurements will take place as part of the MAIN study protocols.

8. ASSESSMENT OF SAFETY

8.1 Specification of Safety Parameters

As per MAIN study

8.2 Definition of an Adverse Event and of a Serious Adverse Event

ADVERSE EVENT

Any untoward medical occurrence in a patient or clinical investigation participant administered a pharmaceutical product and which does not necessarily have a causal relationship with this treatment. An AE can therefore be any unfavorable and unintended sign (including an abnormal laboratory finding), symptom, or disease temporally associated with the use of a product, whether or not related to the product.

SERIOUS ADVERSE EVENT

A serious AE is any untoward medical occurrence that, at any dose:

a) Results in death
b) Is life threatening (the term ‘life-threatening’ in the definition of ‘serious’ refers to an event in which the participant was at risk of death at the time of the event. If does not refer to an event, which hypothetically might have caused death if it were more severe.
c) Required hospitalization or prolongation of an existing hospitalization (hospitalization for elective treatment of a pre-existing condition that did not worsen from baseline is not considered an AE)
d) Results in disability/incapacity
e) Is a congenital abnormality/birth defect.

Use medical and scientific judgment when deciding whether reporting is appropriate in other situations, such as other important medical events that may not be immediately life-threatening or result in death or hospitalization, but may jeopardize the participant or may require medical or surgical intervention to prevent one of the outcomes listed in the above definition.

SUSPECTED UNEXPECTED SERIOUS ADVERSE REACTION (SUSAR)
A suspected unexpected serious adverse reaction is an AE that is determined to be related to the radiotracer and is both serious and unexpected. The term 'unexpected' means that nature or severity of the event is not consistent with the applicable product information (Appendix G.)

8.3 Documentation and classification of study specific adverse events

For regulatory purposes to use the standard radiotracer ^{18}F FDG in this EXPLORATORY STUDY all AEs deemed related to radiotracer administration will be documented in an EXPLORATORY STUDY AE log including diagnosis, severity, toxicity, date of onset and resolution and any action taken. Toxicity will be determined using existing CTCAE version 4.0 criteria, consistent with those proposed for use in the MAIN studies. Severity will be determined based on the following scale:

- Mild: does not interfere with participant's usual function
- Moderate: interferes to some extent with participant's usual function
- Severe: interferes significantly with participant's usual function

All AEs will be recorded in the MAIN study participant file as per MAIN study processes. Where an AE is deemed a result of EXPLORATORY STUDY activities (^{18}F FDG radiotracer administration or other imaging procedures), this will be made clear in documentation and discussed with the MAIN study principal investigator prior to any appropriate escalation to MAIN study medical monitor and sponsor.

8.4 Relationship to Study Investigation

For the purposes of this study for an AE to be considered related to ^{18}F FDG it must occur within 5 half-lives, or 550 minutes of administration. For an AE to be considered related to other imaging procedures, it must occur during the procedure itself.

Any AEs deemed related to the above EXPLORATORY STUDY procedures will be documented in the EXPLORATORY STUDY AE log by one the Investigator or one of his representatives.

EXPECTEDNESS OF SAEs
There are no specific expected SAEs from participation in whole body PET/MRI or dedicated MRI imaging.

Incidental abnormalities detected on imaging will be discussed between the reporting radiologist, study doctor, principal investigator and participant. If required, further review will be arranged by referral to the general practitioner or appropriate specialist. Any incidental findings are expected to be asymptomatic and unpredictable, given the study population of otherwise well, healthy adult volunteers.

There are no commonly reported adverse reactions to radiopharmaceuticals and ^{18}F FDG in the literature (Appendix G). A multicenter review of radiopharmaceutical use (including ^{18}F FDG) administrations found an AE incidence rate of 2.1 per 100 000 administrations [12]. A single case of a probable cutaneous adverse drug reaction has been reported in an individual with multiple existing drug allergies [13]. This did not preclude the patient from repeated dosing. A single case episode of possible anaphylaxis has been reported in the context of the administration of multiple other medications [14]. In both cases symptomatic treatment was effective. Based on the volume of ^{18}F FDG use in the clinical setting, and paucity of published adverse events, the risk of such events is considered to be extremely low.

8.5 Recording and Prompt Reporting of Events

In all cases of AEs, the reporting process used for the MAIN study from which the participant was recruited will be used. If MAIN study procedures are not available recording and reporting of AEs will be in accordance with QIMRCTSOP013 and QIMRCTSOP016.

Any AE deemed related to the administration of ^{18}F FDG or other imaging procedures will be discussed where possible to further clarify causality with the nominated Nuclear Medicine Independent Medical Advisor.

Any SAE deemed related to the administration of ^{18}F FDG or other imaging procedures will be reported to the Sponsor within 24 hours following discussion with the medical monitor and where possible with the nominated Nuclear Medicine Independent Medical Advisor. The TGA and any other relevant authorities will be notified by the Sponsor (or institutionally approved representative) within 72 hours as per existing TGA guidelines.

REPORTING OF PREGNANCY

Female participants of child bearing potential will undergo pregnancy testing as outlined and as per MAIN study protocol. All pregnancy-related policy and procedures will be managed as per MAIN study protocol in addition to exclusion from further EXPLORATORY STUDY participation.

If the MAIN study from which EXPLORATORY STUDY participants only recruits women of non-childbearing potential, no point of care testing or pregnancy monitoring will take place.

8.6 Halting Rules

The investigator(s) reserve the right to halt the study at any point. There are no specific safety findings expected that may prompt suspension or termination of the EXPLORATORY STUDY. In a situation where the MAIN study is halted, the EXPLORATORY STUDY will also be halted as required.

9. QUALITY ASSURANCE

EXPLORATORY STUDY materials and quality assurance related to the imaging of participants and any involvement onsite at HIRF will take place as per existing

HIRF/RBWH processes. Data quality will be considered and described in the presentation of results.

The MAIN study is a registered clinical trial employing an external study monitor responsible for quality assurance with respect to inoculum and participant safety (including pathology testing and clinical examination/investigations.)

10. STATISTICAL CONSIDERATIONS

10.1 Sample Size

This is a pilot investigation to assess the application of advanced medical imaging in subpatent malaria. The population will comprise of eight adults (male and non-pregnant, non-lactating female participants between 18 and 55 years of age, recruited from the IBSM model MAIN study population.

It is expected that this study will be hypothesis generating. There are no pre-existing PET or prospective MRI imaging trials in malaria published in the literature. Data from this pilot investigation may be used in future power calculations if required.

10.2 Statistical Analysis Plan

All data will be presented descriptively. Pre and post inoculation quantitative imaging metrics will be compared with paired T-tests or Mann-Whitney U tests (statistical significance $p<0.05$). Imaging results may be described with respect to demographic, clinical and biochemical data collected as part of the MAIN study.

11. SOURCE DOCUMENTS AND ACCESS

Upon request, the investigator(s)/institution(s) will permit direct access to source data/ documents for trial-related monitoring, audits, HREC review, and regulatory inspection(s) by the Sponsor (or their appropriately qualified delegate) and Regulatory Authorities provided this is feasible from a technical standpoint. Direct access includes examination, analysis, verification and reproduction of records and reports that are important to the evaluation of the study.

12. ADMINISTRATIVE PROCEDURES

ETHICAL CONSIDERATIONS

The amount of blood to be sampled in the study is not considered to be excessive in healthy adult participants. This study will be carried our according to the Declaration of Helsinki, the NHMRC National Statement on Ethical Conduct in Human Research (2007) and the Notes for Guidance on Good Clinical Practice as adopted by the Australian Therapeutic Goods Administration (2000) (CPMP/CH/135/95) and the ICH GCP Guidelines.

ETHICAL REVIEW COMMITTEE

This Protocol will be submitted for approval to QIMR-B HREC, Clinical Trial Protocol Committee. This protocol will then be submitted for executive approval from RBWH HREC. Following this the HIRF Scientific Advisory Committee will review the Protocol and QIMR-B HREC assessment(s) in order to facilitate expedited Site-Specific Approval from the RBWH/Metro North HREC.

Written approvals will be obtained before volunteers are recruited and participants are enrolled. The Investigators will receive all the documentation needed for submitting the present Protocol to the HREC.
It is the responsibility of the Investigator to report study progress to the HREC as required or at intervals not greater than one year.

REGULATORY AUTHORITIES

This is an EXPLORATORY STUDY to run in parallel to an existing MAIN study clinical trial. The investigation uses the application of imaging using a standard radiotracer at standard dosages that is widely used in the clinical setting. This is administered as per existing local institution processes. Due to the unique nature of radiopharmaceuticals, facilities are able to extemporaneously produce and administer FDG onsite for their own purposes. The RBWH Nuclear Medicine department has TGA approval for manufacture of this radiotracer (Appendix F). This radiotracer is not ARTG listed for this facility, but is approved for GMP production and commercial sale from this manufacturer to other facilities within the state of Queensland. Following discussion with the TGA and study sites, this EXPLORATORY STUDY has a CTN registration for the **regulatory purpose** of accessing the standard radiotracer ^{18}F FDG in a manner that ensures prospective record keeping and transparent usage. This includes processes for AE reporting as documented below.

A Nuclear Medicine Independent Medical Advisor has been nominated to ensure participant safety, to provide advice and ongoing review of any SAEs that may be related to EXPLORATORY STUDY activities. No DSMB has been nominated given the low risk nature of this small population EXPLORATORY STUDY. Participants will receive a copy of a current FDA template for ^{18}F FDG product information (Appendix G), as no generic equivalent exists in Australia.

INFORMED CONSENT

Before recruitment and enrolment into the study, each prospective participant must be given a full explanation of the nature and purpose of the study, and a copy of the Participant Information Sheet and Consent Form to review. This form complies with the consent form requirements outlined in the TGA guidelines. Once the essential study information has been provided, and the Investigator is assured that each individual volunteer understands the implications of participating in the study, the participants will be asked to give consent to participate in the study by signing the informed consent form. The consent form shall be signed and dated by the appropriate parties. A notation that written informed consent has been obtained will be made on the participants' MAIN study file. The completed consent forms will be retained by the Investigator and a copy of these will be provided by the Investigator to the participant.

PARTICIPANT RE-IMBURSEMENT

Participants who are found eligible and complete the study will be compensated $160 for their time. This is in addition to MAIN study compensation.

EMERGENCY CONTACT WITH INVESTIGATORS

All participants will be provided with a Participant Information Sheet and Informed Consent Form with contact details of whom to contact in the case of an emergency related to the EXPLORATORY STUDY.

Participants will also be provided with a contact card as per the MAIN study.

NOTIFICATION OF PRIMARY CARE PHYSICIAN
Where it is deemed appropriate by the investigator(s), study doctor and/or reporting radiologist a letter will be sent to the physician stating the nature of the EXPLORATORY STUDY including any adverse events or incidental findings. A copy shall be retained by the study site as part of the MAIN study participant file.

PROTOCOL AMENDMENTS
The Investigator must not modify the Protocol without first obtaining the agreement of QIMR Berghofer in writing. No changes (amendments) to the Protocol may be implemented without prior approval of QIMR Berghofer and the appropriate HREC. If a Protocol amendment requires changes to the Informed Consent Form, the revised Informed Consent Form, prepared by the Investigator, must be approved by the appropriate HREC.

PROTOCOL DEVIATIONS
All protocol deviations must be reported to the principal investigator. Protocol deviations will be assessed for significance by the principal investigator. Those deviations deemed to have a potential impact on the integrity of the study results, patient safety or the ethical acceptability of the trial will be reported to the HREC prior to completion of participant enrollment. Where deviations to the protocol identify issues for protocol review, the protocol may be amended as above.

RETENTION OF OTHER STUDY SPECIFIC SAMPLES
Electronic imaging data will be retained as per HIRF guidelines. No other samples will be collected.

INSURANCE AND INDEMNITY
This EXPLORATORY STUDY is an observational study to run parallel with existing MAIN study clinical trials. There are no specific expected SAEs from participation requiring indemnity, however in the event of an AE that is deemed related to EXPLORATORY STUDY participation (following Investigator and medical monitor review) the sponsor holds No-Fault Compensation for Clinical Trials insurance policy for this study. The site HIRF is part of the RBWH campus.

The MAIN study holds a No-Fault Compensation for Clinical Trials insurance policy.

FINANCIAL DISCLOSURE AND CONFLICT OF INTEREST
Nil conflicts of interest identified or disclosed.

13. DATA HANDLING, RECORD KEEPING AND PUBLICATION POLICY

DATA CONFIDENTIALITY

Only the investigators, designated staff and designated MAIN study staff will have access to information that identifies a study participant.
All MAIN study participants will be assigned a unique identifier.
Participant identifiers in this EXPLORATORY STUDY will be consistent with those used in corresponding MAIN studies.

DATA CAPTURE METHODS
All imaging data will be collected in accordance with site-specific HIRF protocols.

TYPES OF DATA
Imaging data, reports and quantitative imaging metrics will be electronically stored in accordance with HIRF guidelines.

RECORD KEEPING
All study related documents and records are to be retained for a minimum of fifteen years after MAIN study completion. Written agreement from the Sponsor must precede destruction of the same. In cases where HIRF guidelines preclude the storage of source imaging data, data will be transferred to the sponsor for storage.

PUBLICATIONS POLICY

Publication and reporting of results and outcomes of this trial will be accurate and honest, undertaken with integrity and transparency and in accordance with QIMR BERGHOFER's Policy on Criteria for Authorship. Publication of results will be subjected to fair peer-review. Authorship will be given to all persons providing significant input into the conception, design, execution or reporting of the research according to QIMR BERGHOFER Policy on the Criteria for Authorship.

14. LITERATURE REFERENCES

1. Khoury, D.S., et al., *Effect of mature blood-stage Plasmodium parasite sequestration on pathogen biomass in mathematical and in vivo models of malaria.* Infect Immun, 2014. **82**(1): p. 212-20.
2. Dondorp, A.M., et al., *Estimation of the total parasite biomass in acute falciparum malaria from plasma PfHRP2.* PLoS Med, 2005. **2**(8): p. e204.
3. Barber, B.E., et al., *Parasite biomass-related inflammation, endothelial activation, microvascular dysfunction and disease severity in vivax malaria.* PLoS Pathog, 2015. **11**(1): p. e1004558.
4. Lopes, S.C., et al., *Paucity of Plasmodium vivax mature schizonts in peripheral blood is associated with their increased cytoadhesive potential.* J Infect Dis, 2014. **209**(9): p. 1403-7.
5. Anstey, N.M., et al., *Lung injury in vivax malaria: pathophysiological evidence for pulmonary vascular sequestration and posttreatment alveolar-capillary inflammation.* J Infect Dis, 2007. **195**(4): p. 589-96.
6. Cromer, D., et al., *Where have all the parasites gone? Modelling early malaria parasite sequestration dynamics.* PLoS One, 2013. **8**(2): p. e55961.
7. Franke-Fayard, B., et al., *Sequestration and tissue accumulation of human malaria parasites: can we learn anything from rodent models of malaria?* PLoS Pathog, 2010. **6**(9): p. e1001032.
8. Lacerda, M.V., et al., *Postmortem characterization of patients with clinical diagnosis of Plasmodium vivax malaria: to what extent does this parasite kill?* Clin Infect Dis, 2012. **55**(8): p. e67-74.
9. Roth, E., Jr., *Plasmodium falciparum carbohydrate metabolism: a connection between host cell and parasite.* Blood Cells, 1990. **16**(2-3): p. 453-60; discussion 461-6.

10. Yeo, T.W., et al., *Impaired skeletal muscle microvascular function and increased skeletal muscle oxygen consumption in severe falciparum malaria.* J Infect Dis, 2013. **207**(3): p. 528-36.
11. Laothamatas, J., et al., *Transient lesion in the splenium of the corpus callosum in acute uncomplicated falciparum malaria.* Am J Trop Med Hyg, 2014. **90**(6): p. 1117-23.
12. Silberstein, E.B., *Prevalence of adverse reactions to positron emitting radiopharmaceuticals in nuclear medicine. Pharmacopeia Committee of the Society of Nuclear Medicine.* J Nucl Med, 1998. **39**(12): p. 2190-2.
13. Codreanu, I., et al., *Fluorodeoxyglucose-induced allergic reaction: a case report.* J Oncol Pharm Pract, 2013. **19**(1): p. 86-8.
14. Lee, D.Y., et al., *An unusual case of anaphylaxis after fluorine-18-labeled fluorodeoxyglucose injection.* Nucl Med Mol Imaging, 2013. **47**(3): p. 201-4.

- World Medical Association Declaration of Helsinki – Ethical Principles for Medical Research Involving Human Subjects
- NH&MRC National Statement on Ethical Conduct in Human Research (2007).
- Notes for Guidance on Good Clinical Practice – Annotated with TGA Comments (CPMP/ICH/135/95), as adopted by the Australian Therapeutic Goods Administration (July 2000).
- QIMR BERGHOFER Standard Operating Procedures
- QIMR BERGHOFER Statement on Integrity in Research Conduct (check QIMR BERGHOFER Intranet Corporate Info/Regulatory Affairs/ Policies and Procedures for current version and/or date)
