# Supplementary material for: Positron emission tomography and magnetic resonance imaging in experimental human malaria to identify organ-specific changes in morphology and glucose metabolism: A prospective cohort study
Source: PLoS Med. 2021 May 26;18(5):e1003567. doi: 10.1371/journal.pmed.1003567 (PMC8154100; doi:10.1371/journal.pmed.1003567)
Supplement: Attachment — Submitted filename: woodford.pdf [file pmed.1003567.s008.pdf]

This paper reports an experimental study of malaria.

I was asked for a statistical report and I interpret that to include all aspects of the design and conduct of the study. The version I received did not have page numbers. My numbering has the abstract starting on page 2

Points of detail

Page 7 Testing for normality with a planned sample size of 8 is very optimistic. It would have been similarly optimistic to test for any of the other assumptions.

Page 8 I think it might be clearer to state in the caption to Table 1 that it was linear interpolation between measurements on the log scale. At least I assume that is what it means.

Pages 9 to 12 It seems rather unwieldy to report summary statistics for seven observation divided into two groups. This is using six summaries to report seven observations. Why not present the raw data tabulated in a format similar to the supplementary tables and then just describe them in the text? Assuming the authors used t–tests here it might be better to report means with confidence intervals especially when we move to ones where no differences reached the chosen level of statistical significance.

Page 11 I think it might be better to bring all the safety information together in one place. At the moment there are bits in several places.

Figures These are not super clear as they arrived here but I assume the final production process deals with image quality.

Point of more substance

Sample size and tests used

The authors have stated in the methods that they would use either Student's t tests or the appropriate Wilcoxon type test conditional on a preliminary test. For the situation they found themselves in after the withdrawal of one participant they have three in one group and four in the other. For

comparison between groups there are then only

$$\binom{7}{3} = \frac{7!}{3!4!} \quad (1)$$

$$= 35 \quad (2)$$

possibilities. Using the Wilcoxon–Mann–Whitney this means that even the most extreme possible result has two–sided probability $\frac{2}{35} = 0.057$ so we know from the start before we even collect any data that no possible outcome can reach the 0.05 level, or indeed any popular α. Since the authors do report significant group differences they cannot in fact have used such a test and I think they should, for transparency, report what they did do.

For the paired situation the situation is not so extreme as there are 2^7 possibilities so the three most extreme in each direction yield a two–sided p–value $\frac{6}{128} = 0.047$.

This does raise the issue of how much meaning we can extract from the tests where no differences were found and I think we need to be hyper–cautious here. Confidence intervals about the differences would help here as I suggested above. I am not saying there is anything wrong with having small samples and performing statistical analysis on them but we do need to recognise the limitations.

Is this a trial?

There are arguments that this is a trial although it is obviously not of a therapeutic intervention. I am not sure that we need to throw the whole CONSORT rule book at it but there are two things which strike me as important. The authors have reported on masking of outcome assessment and correctly so. What is though lacking is a sample size determination. This does not have to be based on a statistical calculation, it could be based on finance or the availability of equipment, specialised staff or volunteer participants. We just need enough to reassure us that the authors did not stop early for benefit or futility.

Summary

Some clarifications and some more detail about analysis and design.

Michael Dewey
