# Supplementary material for: Positron emission tomography and magnetic resonance imaging in experimental human malaria to identify organ-specific changes in morphology and glucose metabolism: A prospective cohort study
Source: PLoS Med. 2021 May 26;18(5):e1003567. doi: 10.1371/journal.pmed.1003567 (PMC8154100; doi:10.1371/journal.pmed.1003567)
Supplement: Attachment — Submitted filename: ResponseToReviewers.docx [file pmed.1003567.s009.docx]

**PLOS Medicine submission PMEDICINE-D-20-04576R1**

Requests from the editors:

1.	Please address the reviewers’ comments below.

*2.	Please revise your title according to PLOS Medicine's style. Your title must be nondeclarative and not a question. It should begin with main concept if possible. "Effect of" should be used only if causality can be inferred, i.e., for an RCT. Please place the study design ("A randomized controlled trial," "A retrospective study," "A modelling study," etc.) in the subtitle (ie, after a colon).*

The title has been revised according to the PLOS Medicine style - Positron emission tomography and magnetic resonance imaging in experimental human malaria: a prospective cohort study to identify organ-specific changes in morphology and glucose metabolism.

*3.	Abstract:
a.	Please include summary participant demographics (age, sex, etc.).*

These have now been included

*b.	Please include study dates and setting.*

These have now been included

*c.	Please quantify all results with p values and 95% CIs.*

Results have been altered in the text to read mean (95% CI) unless otherwise expressly stipulated. P values included in the text have been edited to refer to the nature of the particular statistical comparison in each case. Asterisks in figures have been altered to include specific P values (matching the text).

*d.	In the last sentence of the Abstract Methods and Findings section, please describe the main limitation(s) of the study's methodology.*

The main limitations (included in the discussion section) have now been reiterated in the abstract.

*e.	Line 54: Please begin with “In this study, we observed that…”*

This has now been included.

*f.      Please include trial registration numbers for the included studies.*

The trial registration numbers of the contributing IBSM studies have been included. Furthermore, we have highlighted in the main body methods section the relationship of the imaging study to the three contributing IBSM trials, and discussed this further in our responses to Reviewer 3.

*4.	At this stage, we ask that you include a short, non-technical Author Summary of your research to make findings accessible to a wide audience that includes both scientists and non-scientists. The Author Summary should immediately follow the Abstract in your revised manuscript. This text is subject to editorial change and should be distinct from the scientific abstract. Please see our author guidelines for more information:* [*https://journals.plos.org/plosmedicine/s/revising-your-manuscript#loc-author-summary*](https://journals.plos.org/plosmedicine/s/revising-your-manuscript#loc-author-summary)

An author summary has now been included between the abstract and introduction of the revised manuscript.

*5.	Methods: Please also include trial registration numbers for the two published studies.*

These have now been included.

*6.	Line 215: Please avoid “approaching statistical significance” – instead state “was not statistically significant”.*

This has been edited, and our data presentation/analysis altered in line with comments from the reviewers.

*7.	Please include as Supporting Information the full protocol for this specific analysis/sub-study, as approved by the ethics committee prior to the start of the study.*

This has now been referred to in the Study Approval section of the Methods, and an additional file SI: imaging study protocol included in the resubmission process.

*8.	Please ensure that the study is reported according to, for example, the STROBE or TREND guidelines (which can be found here:* [*http://www.equator-network.org/*](http://www.equator-network.org/)*), and include the completed checklist as Supporting Information. When completing the checklist, please use section and paragraph numbers, rather than page numbers. Please add the following statement, or similar, to the Methods: "This study is reported as per the ___ guideline (S1 Checklist).*

The statement “This study has been reported as per the STROBE guidelines (S1 Checklist)” has now been included in the Study Approval section of the Methods, and an additional file S1: Checklist included in the resubmission process.

**Comments from the reviewers:**

*Reviewer #1: The authors report a very interesting and singular report using PET imaging to generate evidence of a relatively high degree of tropism for splenic tissues by P. vivax compared to P. falciparum. 

Some concerns:

1) The authors seem to presume readers understand PET imaging technology. The introduction should make clear precisely what is being detected, i.e., increased glucose consumption per se rather than any parasite-specific signal. The greatest uncertainty appears barely acknowledged -- that PET signal from the spleen may not be parasite biomass, but simply downstream physiological responses to the infection unrelated to biomass in a quantitative sense.*

We agree that a limitation of the FDG PET imaging employed in this work, and highlighted in the discussion, is the lack of specificity, i.e. differentiating between parasite- and host-derived response. In the revision we have made further efforts to highlight this in the abstract, introduction and discussion sections. In the introduction we address this by referring to the use of this technology “to localize organ-specific parasite accumulation and host response in life” rather than either process in particular. Furthermore, we highlight that we sought to investigate changes in “glucose metabolism in the spleen, vertebral bone marrow, and liver” rather than specifically parasite or host activity. We have now also included a further clarification of FDG as a radiotracer, outlining that this is a ‘widely available radiotracer that targets glucose utilization.’ A subsequent exploration of the possible causes of changes in radiotracer uptake has been included in the discussion. FDG is the most commonly available radiotracer, and future studies using a potentially more specific agent, though technically very challenging, would be extremely interesting.

*2) The authors cite the specific findings of Barber et al. 2015 (ref.7) that suggested a dominant biomass seated in extravascular spaces, and then express of those, "Nonetheless, this hypothesis is challenging to confirm...". With specific regard to that hypothesis, were Barber et al. the first to propose it? Those authors seem to be point to the CMR review by Baird in 2013 as its origin, as do others.*

Thank you for highlighting this important paper. We agree that the CMR review by Baird provides both historical context and fundamental scientific rationale for the hypothesis that there is a significant extravascular parasite biomass in *P. vivax* infection. As a result we have now prominently cited this in the introduction of the manuscript discussing the relationships between sequestration and disease manifestations, and the possibility of a hidden reservoir of vivax parasites. The findings of Barber et al. quantify the relative discordance between biochemical estimates of endothelial activation and total vivax biomass across a range of clinical severities, which we have used to highlight the challenge of investigating for this phenomenon in life.

*3) There appears to have been a merging error as the legend to Figure 3 does not appear in the PDF. The difference between baseline and post-inoculation appears wholly unremarkable to this reviewer's untrained eye.*

We agree that the differences are subtle, and confirming that they are present will depend on how they are viewed (on a high resolution monitor screen or in the printed version). In accordance with contemporary guidelines on image processing, we have not manipulated the output such as arbitrarily altering contrast. There is a discernible difference between splenic shading at baseline and post-inoculation when the submitted figure is viewed on our workstations. Inclusion of this figure also highlights the methodological novelty of this study to the reader, which is an important aspect of this manuscript. We have reformatted the figure to confirm imaging quality and will ensure that all figure quality checks are repeated prior to re-submission. The figure caption should read: Figure 3: Baseline and post-inoculation PET imaging in Participant 1 (*P. vivax* group). Increased radiotracer uptake is evident in the spleen on post-inoculation imaging (arrowhead).

*4) Reference 3 seems quite important here but is not yet available and, so far as one may now suppose, may not be available in the near future. Are the authors supposing acceptance and full citation by the time this paper gets to press? If so, the details of that investigation should appear in this manuscript. It is not reasonable to cite it as though available, but to withhold the essential findings.*

This reference (now Reference 7 in the revised manuscript) was undergoing review at the time of submission and is currently under internal editorial review. The citation is given in the necessarily concise background section of the manuscript introduction. We have expanded on this a little, but so the essential findings are accessible to the reviewer, we have now uploaded a proof of this manuscript in our resubmission to PLOS medicine. Furthermore, we have added a new reference from the same group (Reference 11 in the revised manuscript) that is currently under review for publication in the vivax issue of PLOS Medicine and made request to the Editors that this related manuscript be made available to reviewers if needed.

*5) The most compelling evidence of extavascular tropism for P. vivax is not described or cited by the authors, appearing to be a significant omission. Specifically, the multiple papers from the laboratories of Bruce Russell, Benoit Malleret, Laurent Renia, and Wei Hong Tham, especially their Science paper of 2018. If P. vivax invasion occurs only in the youngest reticulocytes (and erythroblasts) expressing CD71, an infection of extravascular hemopoietic tissues (like those in the marrow and spleen) may logically be where the infectious biomass is seated. The exclusion of direct molecular evidence in the context of the original hypothesis of extravascular tropism is unfortunate.*

We agree that the 2018 Science paper is critically important in characterizing parasite interactions with reticulocytes and highlights the predilection for younger cells, which is of significant relevance to the hypothesized anatomical location of a hidden compartment. In the revised manuscript, we have now included this paper, and the paper by Malleret et al. 2015 Blood (doi: 10.1182/blood-2014-08-596015) from this group in the introduction, to refer to localization of parasite in organs with CD71 expressing red cells. The Mallaret et al paper raises the hypothesis of a bone marrow niche for hidden biomass compared to the 2018 paper. In contrast to mice, where the spleen is an erythropoietic organ, the spleen in humans is not physiologically a site of erythropoiesis, with splenic erythropoiesis occurring only in rare haematological disease processes (e.g. myelofibrosis). Because the spleen is not physiologically a haematopoietic organ in humans, we do not refer to it as such. Splenic accumulation of immature reticulocytes has not previously been shown in humans. Nevertheless, emerging data (Kho S, et al, PLOS Medicine, under review as a companion paper for the *P. vivax* research special edition) shows that the human spleen physiologically traps less-deformable immature reticulocytes released from the bone merrow, thereby becoming a physiological reservoir for reticulocyte maturation and making the spleen an attractive location for *P. vivax* tropism and replication. We have taken the opportunity to update the discussion with the findings of this PLOS Med edition companion submission, and also more recently published evidence (del Portillo 2020 PNAS) where expression of spleen dependent adhesion molecules is described.

*Reviewer #2: A hot topic in contemporary malaria research is whether there are hidden life cycles of malaria parasites in certain organs. Discussed sites, where hidden life cycles might occur, include the bone marrow and the spleen. However, conclusive experimental data in support of hidden life cycles are scarce. Woodford et al. have now used an elegant in vivo, whole body imaging techniques to detect organs in which the glucose biomimetic [18F] fluorodeoxyglucose accumulates during the course of experimentally induced P. falciparum and P. vivax blood stage infections in human volunteers. The data presented suggest a strong splenic tropism for P. vivax and, to a lesser extent, for P. falciparum, but no accumulation in the bone marrow or the liver. These findings are interpreted in terms of a putative P. vivax replicative cycle in the spleen.
The selling point of the manuscript is the combination of the imaging technique with the experimentally induced blood stage infection in human volunteers. The observations made are potentially of interest, albeit they do not exclude the possibility of hidden life cycles occurring in organs other than the spleen at later time points in the infection, respectively at higher parasitemia.*

Queries:
*1.	This reviewer assumes that the standard uptake value refers to the uptake of [18F] fluorodeoxyglucose. This should be better explained in the manuscript. Can the SUV be converted into absolute molecule numbers?*

It is not possible to convert SUV into an absolute number of molecules, as the SUV represents a unit-less ratio of radioactivity at time of imaging in a given area of interest to the total per kilogram radiation dose administered via radiotracer (in this case FDG). To assist readers less familiar with nuclear medicine, a description of SUV has now been included in the main text methods section. Imaging variables related to SUV estimation have also now been included in the Supplementary text, for those interested in the PET analysis. This includes the calculation of radiotracer uptake rate constants, which are a less commonly used measure of activity (compared to SUV) (S1 and S2 text) and a newly included section discussing how inter-scan variability was minimized, highlighting the rigorous methodology of our imaging protocol (S2 text).

*2.	The different host cell tropism of P. falciparum (mature erythrocytes) and P. vivax (reticulocytes) should be mentioned.*

We have added this to the opening lines of the manuscript, with further reference specifically to vivax reticulocyte specificity, and a possible predilection for reticulocyte-containing organs.

*3.	The number of volunteers is quite small, raising concerns regarding the statistical significance of the findings.*

We agree that the small number of subjects, inherent in a study of this type, is a limitation, and have highlighted this in the methods section (regarding statistical comparisons). We have also discussed this in the limitations section of the manuscript. Furthermore, edits in data presentation and analysis have been made in keeping with the advice of Reviewer 3 (statistical reviewer) to ensure that this concern is addressed.

*4.	Is the enhanced splenic activity due to replicating parasites or is it a reflection of increased immunological activity, e.g., by macrophages present in the splenic cord, which remove lysed infected reticulocytes and/or pitted parasites following their destruction at the splenic slits?*

Although this is a key observation, we are unable to confirm the relative contribution of these possibilities using this imaging modality. As a result, each possibility has been explored in the discussion, and we have modified the introduction to highlight that glucose utilization is the outcome metric of FDG PET imaging. Irrespective of the underlying cause of increased utilization, we present that glucose uptake is greater in the spleens of vivax infected individuals, compared to that in *P. falciparum* infection.

*5.The text could be substantially shortened and the number of display items could be reduced to two.*    

Although we have now removed some discussion, we have had difficulty in significantly shortening the manuscript, especially in respect to addressing the comments and recommendations of the Editors and Reviewers. If the Editor requires that the display be reduced, we would be prepared to shift Fig 1 (parasitemia curves) to the Supplementary Information section. In this revision we have left it as is, as these curves visually outline the course of infection prior to imaging.

Reviewer #3: *Comments copied from PDF attachment*
Points of detail

*Page 7: Testing for normality with a planned sample size of 8 is very optimistic. It would have been similarly optimistic to test for any of the other assumptions.*

We agree that the sample size is small and that the presented statistical analysis is optimistic. We have revised the Methods and Results sections in keeping with your comments, and those of the Editors and other reviewers, and made the following changes:

- Mean and 95% CI is now presented as the summary statistic of choice
- Effect sizes have been removed
- Commentary regarding normality testing has been removed and instead the nature of the specific statistical comparisons has been incorporated. This includes describing specific tests adjacent to where p values presented in the text and figures

*Page 8: I think it might be clearer to state in the caption to Table 1 that it was linear interpolation between measurements on the log scale. At least I assume that is what it means.*

We have edited the Table 1 caption to reflect this recommendation.

*Pages 9 to 12: It seems rather unwieldy to report summary statistics for seven observation divided into two groups. This is using six summaries to report seven observations. Why not present the raw data tabulated in a format similar to the supplementary tables and then just describe them in the text? Assuming the authors used t-tests here it might be better to report means with confidence intervals especially when we move to ones where no differences reached the chosen level of statistical significance.*

We acknowledge that summary statistics for the study population do not well summarize the available data, and considered this during the initial drafting of the manuscript. However, in an effort to provide all information to readers, we elected to make both summary statistics, and the raw available to readers. The mean and 95% CI has now been included as the summary of choice throughout the manuscript.

*Page 11: I think it might be better to bring all the safety information together in one place. At the moment there are bits in several places.*

We now have moved the imaging safety material initially presented early in the results to a new section “Safety, clinical review and laboratory parameters” to bring all of this information together as suggested.

*Figures: These are not super clear as they arrived here but I assume the final production process deals with image quality.*

We apologize for the suboptimal image quality in the version available for review. All images have been re-formatted and will be re-checked using the PLOS image assessment software prior to resubmission.

Point of more substance

*Sample size and tests used*

*The authors have stated in the methods that they would use either Student's t tests or the appropriate Wilcoxon type test conditional on a preliminary test. For the situation they found themselves in after the withdrawal of one participant they have three in one group and four in the other. For comparison between groups there are then only <formula> possibilities. Using the Wilcoxon-Mann-Whitney this means that even the most extreme possible result has two-sided probability 2/35 = 0.057 so we know from the start before we even collect any data that no possible outcome can reach the 0.05 level, or indeed any popular alpha. Since the authors do report significant group differences they cannot in fact have used such a test and I think they should, for transparency, report what they did do. For the paired situation the situation is not so extreme as there are 2^7 possibilities so the three most extreme in each direction yield a two-sided p-value 6/128 = 0.047. This does raise the issue of how much meaning we can extract from the tests where no differences were found and I think we need to be hyper-cautious here. Confidence intervals about the differences would help here as I suggested above. I am not saying there is anything wrong with having small samples and performing statistical analysis on them but we do need to recognise the limitations.*

We agree that the sample size is small and that the presented statistical analysis is optimistic. Due to the novel nature of the study, we wish to present as much of an analysis as possible. In the Methods section, we refer to the statistical comparisons as an ‘exploratory analysis’ in an effort to outline these limitations. We have now added a further acknowledgement of this to the first paragraph of the Discussion, and have elaborated on this further in the limitations section.

*Is this a trial?*

*There are arguments that this is a trial although it is obviously not of a therapeutic intervention. I am not sure that we need to throw the whole CONSORT rulebook at it but there are two things which strike me as important. The authors have reported on masking of outcome assessment and correctly so. What is though lacking is a sample size determination. This does not have to be based on a statistical calculation, it could be based on finance or the availability of equipment, specialised staff or volunteer participants. We just need enough to reassure us that the authors did not stop early for benefit or futility.*

As would be appreciated, nesting these imaging studies within approved clinical trials was the only means available to do this work. This inevitably posed some governance challenges (subjects cannot be simultaneously enrolled in two clinical trials). After taking this advice, we established this study as a prospective cohort study nested within the relevant clinical trials. We did not consider the use of an imaging test commonly used in clinical medicine as an ‘experimental test.’ We have now completed a STROBE checklist as requested by the Editors. The title has been formatted to clarify the nature of the study, in keeping with PLOS guidelines. Furthermore, we have added extra detail to the Methods section regarding study registration and the relationship of the imaging study to the contributing IBSM studies. The IBSM studies were clinical trials, with 3 separate IBSM studies contributing subjects to the imaging study presented in this manuscript. In two IBSM studies, the imaging activities were performed as a part of a standalone sub-study protocol linked to the relevant IBSM study. In the third IBSM study, which was under the sponsorship of our institution, imaging activities were incorporated into IBSM study protocol as an optional exploratory component (with separate consent). Study registration numbers for all relevant protocols have been included in the manuscript. Due to novel nature of the study, multiple institutions involved, and different study sponsorship between IBSM studies, it was not possible to undertake this study under a single umbrella protocol. While we agree that this could generate some confusion, and in an ideal world it would have been undertaken as a freestanding protocol, the ethical and logistic challenges to do so would have been formidable. We therefore consider that presentation of the pooled data for subjects that took part in the imaging study, where in all cases subjects underwent identical imaging procedures with the approval of relevant institutional review boards, is an acceptable approach.

We have removed the wording regarding imaging reporting and IBSM inoculum species and the word ‘blinded’ to reduce possible reader confusion regarding study design. Nonetheless we believe that it s important to convey to the reader that the interpretation and reporting of the key study endpoint (the imaging) was undertaken in a blinded fashion. In keeping with the suggestions, we have included a sentence regarding the planned enrollment of 8 individuals in the first paragraph of the Methods section – this was based on the number of available studies able to contribute subjects, and availability of imaging facilities occurring during the period for which funding was available.

Reviewer #4: General comments:
*This is an interesting pilot study demonstrating the value of performing imaging in controlled human malaria infection studies. Despite the fact that the number of volunteers in the study is extremely small, the pioneering nature of the research should be applauded. 

However, the main conclusion of the manuscript "greater early splenic tropism in Plasmodium vivax than Plasmodium falciparum infection" seems to be driven primarily by a significant different change in SUV between Pf and Pv inoculated volunteers. Here, there seems to be an error in the analysis. Line 216 states that splenic SUV for the Pv group increases a median of 15.9% (range 8.8-21.8%) whereas line 227 states that splenic SUV in the Pv group increased a median of 27.6% (range 18.0-40.8%). Given this discrepancy, it seems that, comparing with data from table S1, the data from the splenic volume change in the Pv group (median 27.6%) have been accidentally swapped with the data from splenic SUV change (median 21.8%). This should be corrected. Preferably, the statistical tests used for these data should be listed in the results section.*

Thank you for identifying this error. We can confirm that the unpaired t-test comparison of change in SUV for the *P. vivax* group versus the *P. falciparum* group has a p value of 0.036 and that the error identified was an issue in transcription of results into text format only. We have subsequently revised the presentation and analysis of the results in line with the recommendations of the Editors and Reviewer panel at large and re-checked the figures presented. This includes presentation of results as mean and 95% CI, and describing the specific statistical comparison used adjacent to where p values are presented.

*Looking at the graphs presented in figure 2, it seems to me that the difference between splenic SUV change in Pf and Pv inoculated volunteers is mainly driven by one volunteer in the Pf group who had a high baseline SUV and thus did not further increase. Given the high baseline SUV, this volunteer might significantly bias data.*

The baseline absolute SUV in any individual should not particularly affect subsequent imaging uptake values (or the potential for change in uptake) in the ranges that we have reported. We would also highlight that the relative change in splenic SUV each of the vivax inoculated participants were higher than that for any falciparum inoculated participants, and none of the falciparum group were observed to experience an increase in uptake from baseline >10%, a value that we pre-specified which we have set as the threshold for meaningful change. As a result, the impact of the baseline splenic SUV result in Participant 5 does not alter our interpretation of the results. As noted above, we have now included further description of the nature of SUV as a measurement in the main text methods section, and a discussion of SUV variability in S2 text. We believe that these additions, along with changes in data presentation described above should address concerns regarding bias and the statistical rationale.

*Furthermore, I feel that the clinical review and laboratory parameters could have included more data than just the subset of volunteers for which scans were available. It does not make sense to exclusively present clinical and laboratory data from only the 7 individuals in this paper only, whilst much more data is available from the referenced studies. I would recommend expanding this section to include more data from all participants in the studies.*

Although this analysis is of undoubted interest we believe that it is beyond the scope of this work and including it would substantially increase the length of this manuscript and dilute its central message. An analysis of changes in hemoglobinƒtham data from a large group (n=315) of participants in our challenge studies has been completed and will be submitted as a separate publication. We have recently published a review of safety issues in malaria challenge studies (Odedra AJTMH 2020) and a series of manuscripts where analysis of LFT changes following vivax and falciparum infections have been discussed (Woodford AJTMH 2018; Chughlay AKTMH 2020, Odedra AJTMH 2020).

*Lastly, a minor comment regarding the liver enzyme elevations which were detected. Given the timelapse between imaging and enzyme abnormalities (1-2 weeks), it does not seem justified to draw any conclusions on the etiology based on imaging and enzymes correlations.*

Given the significant interval between PET imaging and enzyme changes (1-2 weeks), and the fact that we have reported these changes in several studies where PET scanning was not undertaken we believe that the imaging was not the cause of these changes. We have included a short discussion regarding elevated LFTs following treatment, as this was observed in several participants. As these LFT elevations are not related to imaging (and vice versa) we have edited the relevant discussion section to make this clearer.
